# Two Secreted Proteoglycans, Activators of Urothelial Cell–Cell Adhesion, Negatively Contribute to Bladder Cancer Initiation and Progression

**DOI:** 10.3390/cancers12113362

**Published:** 2020-11-13

**Authors:** Vasiliki Papadaki, Ken Asada, Julie K. Watson, Toshiya Tamura, Alex Leung, Jack Hopkins, Margaret Dellett, Noriaki Sasai, Hongorzul Davaapil, Serena Nik-Zainal, Rebecca Longbottom, Makoto Nakakido, Ryo Torii, Abhi Veerakumarasivam, Syuzo Kaneko, Mandeep S. Sagoo, Gillian Murphy, Akihisa Mitani, Kohei Tsumoto, John D. Kelly, Ryuji Hamamoto, Shin-ichi Ohnuma

**Affiliations:** 1 UCL Institute of Ophthalmology, University College London, 11-43 Bath Street, London EC1V 9EL, UK; vpapadaki@fleming.gr (V.P.); toshiya.tamura@axcelead.com (T.T.); alex.leung.18@ucl.ac.uk (A.L.); jack.hopkins.18@ucl.ac.uk (J.H.); m.dellett@ulster.ac.uk (M.D.); noriakisasai@bs.naist.jp (N.S.); hd410@cam.ac.uk (H.D.); e.longbottom@nsh.net (R.L.); nakakido@protein.t.u-tokyo.ac.jp (M.N.); m.sagoo@ucl.ac.uk (M.S.S.); 2 Institute of Fundamental Biological Research, Biomedical Sciences Research Center “Alexander Fleming”, 16672 Vari, Greece; 3 Division of Molecular Modification and Cancer Biology, National Cancer Center Research Institute, 5-1-1 Tsukiji, Chuo-ku, Tokyo 104-0045, Japan; ken.asada@riken.jp (K.A.); sykaneko@ncc.go.jp (S.K.); 4 Cancer Translational Research Team, RIKEN Center for Advanced Intelligence Project, 1-4-1 Nihonbashi, Chuo-ku, Tokyo 103-0027, Japan; 5 The Hutchison/MRC Research Centre, Department of Oncology, University of Cambridge, Hills Road, Cambridge CB2 2XZ, UK; julie.watson@stemcell.com (J.K.W.); abhiv@sunway.edu.my (A.V.); gm290@cam.ac.uk (G.M.); j.d.kelly@ucl.ac.uk (J.D.K.); 6 Cancer Research UK Cambridge Institute, University of Cambridge, Cambridge CB2 0RE, UK; 7 Stem Cell Technologies, Building 7100, Cambridge Research Park, Beach Drive, Waterbeach, Cambridge CB25 9TL, UK; 8 Integrated Biology, Research Division Axcelead Drug Discovery Partners, Inc. 26-1, Muraoka-Higashi 2-chome, Fujisawa, Kanagawa 251-0012, Japan; 9 C-TRIC, Altnagelvin Hospital Campus, NI Centre for Stratified Medicine, Glenshane Road, Derry/Londonderry BT47 6SB, UK; 10 Developmental Biomedical Science, Graduate School of Biological Sciences, Nara Institute of Science and Technology, 8916-5, Takayama-cho, Ikoma 630-0192, Japan; 11 Cambridge Biomedical Campus, Jeffrey Cheah Biomedical Centre, Puddicombe Way, Wellcome—MRC Cambridge Stem Cell Institute, Cambridge CB2 0AW, UK; 12 MRC Cancer Unit University of Cambridge Hutchison/MRC Research Centre, Box 197, Cambridge Biomedical Campus, Cambridge CB2 0XZ, UK; snz@mrc-cu.cam.ac.uk; 13 Academic Laboratory of Medical Genetics, Box 238, Lv 6 Addenbrooke’s Treatment Centre, Addenbrooke’s Hospital, Cambridge CB2 0QQ, UK; 14 Critical Care, University College London Hospital, 3rd floor, 235 Euston Road, London NW1 2PG, UK; 15 Institute of Medical Science, The University of Tokyo, 4-6-1 Shirokanedai, Minato-ku, Tokyo 108-8639, Japan; tsumoto@bioeng.t.u-tokyo.ac.jp; 16 Department of Mechanical Engineering, University College London, Torrington Place, London WC1E 7JE, UK; r.torii@ucl.ac.uk; 17 Department of Biological Sciences, School of Science and Technology, Sunway University, Bandar Sunway, Selangor Darul Ehsan 47500, Malaysia; 18 Retinoblastoma Service, Royal London Hospital, Whitechapel Road, London E1 1BB, UK; 19 Ocular Oncology Service, Moorfields Eye Hospital, City Road, London EC1V 2PD, UK; 20 National Institute for Health Research Biomedical Research Centre at Moorfields Eye Hospital NHS Foundation Trust and UCL Institute of Ophthalmology, City Road, London EC1V 2PD, UK; 21 Department of Respiratory Medicine, The University of Tokyo Hospital, 7-3-1 Hongo, Bunkyo-ku, Tokyo 113-8655, Japan; mitania-tky@umin.ac.jp; 22 Division of Surgery and Interventional Science, University College London, 74 Huntley Street, London WC1E 6AU, UK

**Keywords:** OMD, PRELP, tumor suppression gene, bladder cancer initiation, tight junction, partial EMT

## Abstract

**Simple Summary:**

Epithelial–mesenchymal transition (EMT) is associated with cancer progression. Here, we found that two secreted proteins of osteomodulin (OMD) and proline/arginine-rich end leucine repeat protein (PRELP) were selectively expressed in bladder umbrella epithelial cells, and they were suppressed in bladder cancer. We revealed that *OMD*^−/−^ or *PRELP*^−/−^ knockout mice caused a breakdown of the umbrella cell layer through weakening cell–cell integrity and the activation of partial EMT, which resulted in the formation of early bladder cancer-like structures, while OMD or PRELP application to bladder cancer cells inhibited cancer progression through reversing EMT, which was mediated by the inhibition of TGF-β and EGF. Our result indicates that OMD and PRELP function as tumor-suppressing proteins through inhibiting EMT. OMD and PRELP may be potential therapeutic targets in bladder cancer.

**Abstract:**

Osteomodulin (OMD) and proline/arginine-rich end leucine repeat protein (PRELP) are secreted extracellular matrix proteins belonging to the small leucine-rich proteoglycans family. We found that OMD and PRELP were specifically expressed in umbrella cells in bladder epithelia, and their expression levels were dramatically downregulated in all bladder cancers from very early stages and various epithelial cancers. Our in vitro studies including gene expression profiling using bladder cancer cell lines revealed that OMD or PRELP application suppressed the cancer progression by inhibiting TGF-β and EGF pathways, which reversed epithelial–mesenchymal transition (EMT), activated cell–cell adhesion, and inhibited various oncogenic pathways. Furthermore, the overexpression of OMD in bladder cancer cells strongly inhibited the anchorage-independent growth and tumorigenicity in mouse xenograft studies. On the other hand, we found that in the bladder epithelia, the knockout mice of OMD and/or PRELP gene caused partial EMT and a loss of tight junctions of the umbrella cells and resulted in formation of a bladder carcinoma in situ-like structure by spontaneous breakdowns of the umbrella cell layer. Furthermore, the ontological analysis of the expression profiling of an OMD knockout mouse bladder demonstrated very high similarity with those obtained from human bladder cancers. Our data indicate that OMD and PRELP are endogenous inhibitors of cancer initiation and progression by controlling EMT. OMD and/or PRELP may have potential for the treatment of bladder cancer.

## 1. Introduction

Small leucine-rich proteoglycans (SLRPs) are a family of 17 secreted extracellular matrix (ECM) proteoglycans [1]. SLRP members function not only as modifiers of ECM organization but also as regulators of ligand-induced signaling pathways [1,2,3,4]. For example, Tsukushi regulates the Notch, Wnt, FGF, BMP4, and Nodal pathways through interactions with extracellular components in a context-dependent manner [5,6,7,8]. The expression of SLRPs is often altered in tumors. Biglycan, lumican, and fibromodulin are overexpressed in various types of cancer, whilst decorin is overexpressed in some types of cancer and suppressed in others [9]. High expression levels of lumican are associated with a poorer survival in colorectal tumors, and they are also presented with increased metastasis in lung cancers [10]. Conversely, the overexpression of lumican in melanoma cells inhibited tumor formation in an animal model [11], whereas low expression levels of lumican and decorin are associated with a poorer patient survival in breast tumors and spindle cell carcinomas, respectively [12]. Thirty percent of decorin knockout mice develop intestinal tumors [13], and decorin/p53 double knockout mice demonstrate an enhanced susceptibility to thymic lymphoma [14]. Decorin suppressed squamous cell carcinoma in vitro by binding to EGFR to regulate downstream signaling pathways, while it also inhibited tumor formation and metastasis in a xenograft model [1,15,16]. However, no mutations or deletions of these genes have been reported so far in human cancers. Thus, their relevance to human carcinogenesis remains unclear.

With the development of epithelial malignancies, major changes occur in the organization of ECM, which normally provides the microenvironment for the maintenance of epithelial cell integrity. Many oncogenes cannot initiate a tumor if the extracellular microenvironment is normally maintained [17]. Moreover, in some cases, breakdown of the extracellular microenvironment by itself can trigger tumorigenesis [18]. These studies further demonstrate the importance of ECM proteins in cancer development.

Bladder cancer is one of the most common cancers worldwide, with 549,400 new cases and 200,000 deaths annually [19]. Our study shows that the two SLRPs or secreted ECM, osteomodulin (OMD) and proline/arginine-rich end leucine repeat protein (PRELP) are expressed in bladder and critical regulators of bladder cancer initiation and progression via altering cell–cell adhesion, probably through the regulation of epithelial–mesenchymal transition (EMT). Our findings can explain the mechanism of cancer initiation and can contribute to new therapeutic applications.

## 2. Results

### 2.1. OMD and PRELP Expression and the Association with the Early Stages of Bladder Cancer

**Figure 1:**
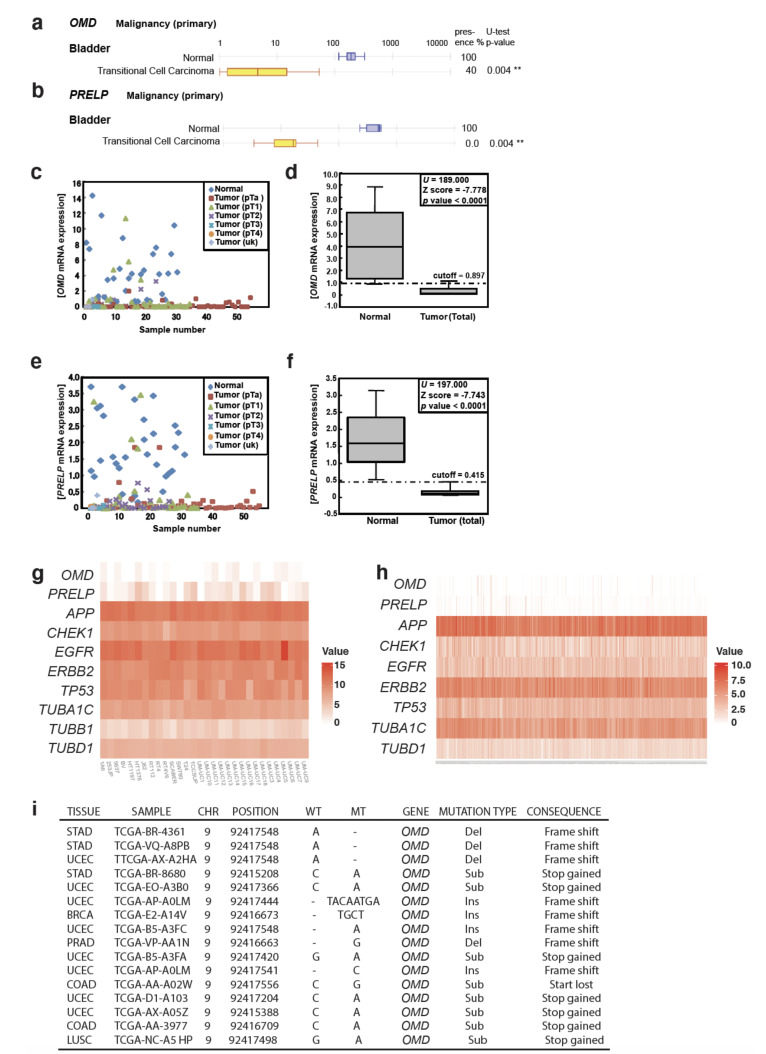
Expression of osteomodulin (OMD) and proline/arginine-rich end leucine repeat protein (PRELP) in cancer (**a**,**b**). Microarray analysis of *OMD* (**a**) and *PRELP* (**b**) expression in human bladder cancer samples and normal bladder tissues. (**c**) Quantitative analysis of *OMD* expression in bladder cancer at different stages by qPCR. (**d**) Box–whisker plot (median 50% boxed) of (**c**). Cutoff value (dash line) was determined as described in Materials and Methods. (**e**) Quantitative analysis of *PRELP* expression in bladder cancer at different stages by qPCR. (**f**) Box–whisker plot of (**e**). (**g**) Expression analysis of *OMD* and *PRELP* in bladder cell lines. Published expression profiling data of MIBC cell lines (GSE97768) are re-examined to elucidate the relative expression of *OMD* and *PRELP* in comparison with known overexpressing genes in bladder cancer; *APP*, *CHEK1*, *EGFR*, *ERBB2*, and *TP53* and with housekeeping genes of *TUBA1C*, *TUBB*, and *TUBD1*. (**h**) Expression analysis of *OMD* and *PRELP* in bladder tissues samples from patients. Published expression profiling data of non- Non-muscle-invasive bladder cancer (NMIBC)(E-MTAB-4321) are re-examined to elucidate the relative expression of *OMD* and *PRELP* in comparison with *APP*, *CHEK1*, *EGFR*, *ERBB2*, and *TP53* and with housekeeping genes of *TUBA1C* and *TUBD1*. Details of both (**g**,**h**) analyses are in Materials and Methods. (**i**) Somatic mutations in human cancer samples that are predicted to generate a loss of function of *OMD*. Detail of cancers is described in Materials and Methods in the section of OMD and PRELP Expression Analysis in muscle-invasive bladder cancer (MIBC) cell lines and NMIBC Patient Samples. ** indicates *p* < 0.01.

We analyzed the expression levels of SLRP members in various epithelial cancers including bladder cancer using two independent microarray-based expression-profiling databases drawn from a worldwide population: Oncomine (Figure 1a,b; Figure S1) and Gene Logic Inc (Figure S2). Interestingly, the expression levels of *OMD* and *PRELP* are strongly suppressed in the majority of epithelial cancer types.

Next, we performed a detailed expression analysis of 126 bladder cancer samples and 31 normal control samples (Figure 1c–f; Table S1). The expression of both *OMD* and *PRELP* in tumors was drastically lower compared to normal tissues (Figure 1d,f) and declined progressively with cancer stage (Figure 1c,e; Table S1). No associations were found with gender or recurrence status, nor with age or tumor size (Table S1). *OMD* and *PRELP* were also downregulated in bladder cancer cell lines compared to normal bladder tissue (Figure S3a,b). Moderate *OMD* expression was seen only in the non-invasive bladder cell lines RT4 and LHT1376.

To assess the potential role of OMD and PRELP as diagnostic markers, we set cutoff values to distinguish tumor samples from normal tissues through calculation of the interquartile range. The expression levels of *OMD* and *PRELP* in almost all normal bladder tissues were above the cutoff value (specificity: 83.9% (*OMD*) and 90.3% (*PRELP*)), while expression in the vast majority of tumor tissues was below the cutoff (sensitivity: 88.9% (*OMD*) and 90.5% (*PRELP*), Table S2). Expression levels of *OMD* and *PRELP* in the Ta (early) stage of almost all tumor tissues were below the cutoff value (sensitivity: 88.9% (OMD) and 88.9% (PRELP), Table S2). When we combined the data for *OMD* and *PRELP,* the expression of both genes below the cutoff value was found only in tumor samples and in none of the normal tissues (specificity 100%). These results show that the expression levels of *OMD* and *PRELP* genes are powerful markers for the prediction of the presence of urothelial carcinomas. The suppression of *OMD* and *PRELP* was also observed when we analyzed previously published expression profiling data for muscle-invasive bladder cancer (MIBC) and non-muscle-invasive bladder cancer (NMIBC) (Figure 1g,h) [20,21]. An examination of mutation analysis using The Cancer Genome Atlas (TCGA) (Figure 1i) found a total of 3,142,246 somatic substitutions/indels were interrogated from 33,096 primary human cancers, and the somatic mutations predicted to generate a loss-of-function effect in OMD are summarized in Figure 1i. However, relatively few mutations were observed (95 for OMD and 158 for PRELP) (unpublished data).

### 2.2. Cell–Cell Adhesion and Cancer Signaling Regulated by OMD and PRELP

To further assess the role of OMD and PRELP in cancer, we overexpressed or underexpressed the two proteins in cultured cells and performed gene expression analysis using microarrays (Affimetrix GeneChip^®^ System). The T-Rex-293T system was used to express the genes at a near-physiological level without causing adverse effects due to their insertion site. To ablate gene expression, 5637 bladder cancer cells, expressing *OMD* and *PRELP* at a low level, were transfected with siRNA constructs for *OMD* or *PRELP*. After validating the altered expression of *OMD* and *PRELP* by RT-PCR, gene expression profiling was performed.

**Figure 2:**
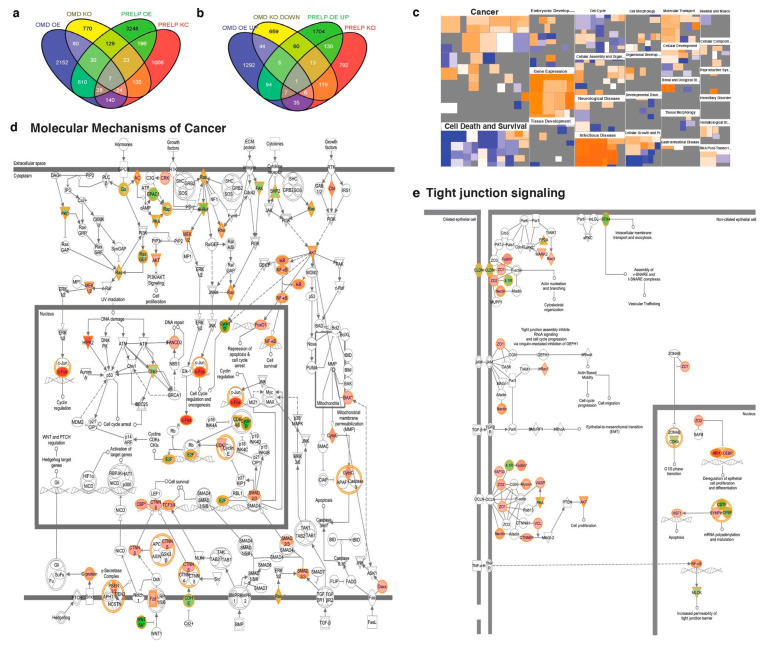
Gene expression profiling in OMD/PRELP overexpressing or deleted cells. Gene expression profiling was performed under seven conditions; OMD overexpression in T-Rex-293T cells, PRELP overexpression in T-Rex-293T cells, control T-Rex-293T cells, OMD depletion in the 5637 bladder cancer cells, PRELP depletion in the 5637 cells, two controls of the 5637 cells. Details are in Materials and Methods. Then, genes with statistical significant changes of mRNA levels have been identified. Data were analyzed, and the following figures were made through the use of Ingenuity Pathway Analysis (IPA) (QIAGEN Inc., https://www.qiagenbioinformatics.com/products/ingenuitypathway-analysis). (**a**) Gene numbers significantly inhibited by OMD overexpression or PRELP overexpression but activated by OMD depletion or PRELP depletion. (**b**) Gene numbers activated by OMD overexpression or PRELP overexpression but suppressed by OMD depletion or PRELP depletion. (**c**) Heat map of signaling pathways significantly affected by OMD overexpression. This heat map was created using IPA software. Similar heat maps were observed in other three conditions of OMD depletion, PRELP overexpression, and PRELP depletion. (**d**,**e**) Schematic drawing of the most strongly influenced biological events regulated by OMD overexpression. “Molecular Mechanism of Cancer” (**d**) and “Tight junction signaling” (**e**) category. Both images of (**d**,**e**) were created by Ingenuity Pathway Analysis according to their rule. This pathway is one of the most strongly influenced ones by any of four conditions (OMD overexpression, OMD depletion, PRELP overexpression, PRELP depletion).

Figure 2a,b show the numbers of genes that are negatively and positively transcriptionally regulated by OMD and/or PRELP, respectively. The genes affected by OMD and PRELP include many oncogenes and tumor-suppressor genes such as *NF*-kB, *Ras*, and *c-Fos*. For example, 107 genes were activated by both OMD and PRELP overexpression, while 139 genes were suppressed by the double-depletion (Figure 2b). These observations indicate that OMD and PRELP have a functional redundancy while they also regulate various distinct target genes.

Next, to elucidate the affected signaling pathways, biological events, and mechanisms, the gene expression profiling data were analyzed with a data mining program (Ingenuity Pathway Analysis, IPA, Qiagen, (https://digitalinsights.qiagen.com/products-overview/discovery-insights-portfolio/analysis-and-visualization/qiagen-ipa/?cmpid=QDI_GA_IPA&gclid=CjwKCAiAtK79BRAIEiwA4OskBpDKfEsg5CJdSERKm3IEd_0gZRXNEGfgu7XJjKoC9hVggrFtzQnvxBoCY_wQAvD_BwE). Using the Functional Analysis mode, “molecular mechanism of cancer” was identified as one of the most significantly affected biological functions and/or diseases in all four conditions of OMD overexpression, OMD depletion, PRELP overexpression, and PRELP depletion (Figure 2c; Figure S3c–f). In total, 304 and 388 genes related to the “cancer” category are significantly affected by the altered expression of OMD and PRELP, including members of the p53 pathway, the NF-kB pathway, the Ras pathway, the RB1 pathway, the Jun/Fos pathway, and the Myc pathway (Figure 2d).

Our analysis also revealed that both OMD and PRELP strongly influence cell–cell adhesion mediated by tight junctions (Figure 2e). Tight junctions are a type of cell–cell junction that binds the apical sides of epithelial cells. The breakdown of tight junctions has been proposed as a critical step in cancer initiation [22,23]. Tight junction components such as Zonula occlugens-1 (ZO-1) and Nectin were transcriptionally activated by OMD or PRELP overexpression, while they were suppressed in OMD or PRELP depletion, suggesting that OMD and PRELP have the ability to positively regulate tight junctions (Figure 2e).

### 2.3. OMD or PRELP Overexpression in EJ28 Bladder Cancer Cells

To investigate the roles of OMD and PRELP at the molecular level, we constructed stable cell lines that overexpressed OMD, OMD-myc, PRELP, and PRELP-myc using the EJ28 bladder cancer cell line, as their endogenous expression is strongly suppressed.

**Figure 3:**
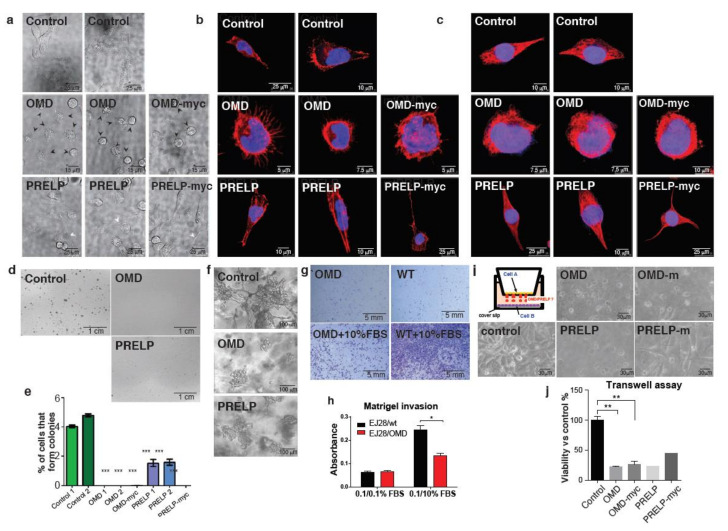
Effect of OMD or PRELP overexpression in bladder cancer cell lines. (**a**) Cell morphology of EJ28 bladder cancer cells transfected with OMD, OMD-myc, PRELP, or PRELP-myc constructs, observed by differential interference contrast (DIC) microscope. Round cells are indicated as arrowheads. (**b**) Phalloidin staining of the transfected EJ28 cells. Phalloidin (red) and DAPI (blue). Pin-like structures of OMD overexpressing cells are phalloidin-positive. PRELP overexpression results in clear long actin fiber formation. (**c**) Anti-tubulin antibody staining. Tubulin (red) and 4′,6-diamidlino-2-phenylindole (DAPI) (blue). (**d,e**) Anchorage-independent growth using the soft agar. Photos of control, OMD, and PRELP overexpressing colonies formed in the top agar layer (**d**). Quantification of the cell percentage that formed colonies (**e**). (**f**) Cell growth in the Matrigel. (**g**,**h**) Cell migration and invasion assay using the Boyden chamber. Photos of cells that invaded to the bottom side of membrane after the addition of fetal bovine serum (FBS) as a chemoattractant (**g**). Quantification of cell migration/invasion in (**g**,**h**). (**i**,**j**) Transwell co-culture assay to evaluate the effect of secreted OMD/PRELP on non-contacting cells. Schematic drawing of the assay system and photos of EJ28 cells cultured at the button chambers (**i**). Quantification of viable cell density in the bottom well by trypan blue staining (**j**). *, **, *** indicate *p* < 0.01, *p* < 0.005, *p* < 0.001, respectively.

Under standard cell culture using non-coated culture dish with non-confluent conditions, control EJ28 cells had a flattened fibroblast-like shape. In contrast, many OMD overexpressing cells had a markedly different round shape with many pin-like extensions (Figure 3a). PRELP overexpression also resulted in a change of cell morphology to round cells similar to OMD overexpressors together with elongated cells with protruding stress fiber-like filamentous extensions (Figure 3a). To evaluate relevant changes in the cytoskeletal structure, we stained for actin and tubulin. We found that there are many pin-like actin structures on the surface of the round OMD-expressing cells, similar to the phenotype induced by cdc42 activation [24]. On the other hand, PRELP overexpression resulted in both round cells with pin-like structures and elongated cells with long clear actin fibers (Figure 3b). These abnormal morphological changes were also observed with tubulin staining (Figure 3c).

We next analyzed the effect of OMD/PRELP overexpression on cell proliferation and survival. First, expression levels of *OMD* and *PRELP* in EJ28 cells were analyzed by qRT-PCR (S4a and b) and by Western blotting using the myc antibody for myc-tag protein expression (S8n and S8p). OMD and OMD-myc cells exhibited reduced proliferation, both in standard proliferation and BrdU incorporation assays (Figure S4c,d), while cell cycle analysis by flow cytometry revealed an enhanced G1 phase transition (Figure S4e). Finally, OMD and OMD-myc cells presented a small but significant increase in apoptosis, as assayed by annexin staining (Figure S4f). The overexpression of OMD, OMD-myc, and PRELP resulted in a slight but significant suppression of cell growth with modulation of the cell cycle phase distribution. In addition, OMD and PRELP overexpression slightly increased apoptosis, although the majority of cells remained non-apoptotic. Overall, we conclude that the overexpression of OMD and PRELP proteins results in a subtle but significant suppression of cell growth with the modulation of cell cycle phase distribution.

Anchorage-independent growth is a well-established property of transformed cancer cells. Therefore, we examined the effect of OMD or PRELP overexpression on anchorage-independent growth (Figure 3d,e). OMD or PRELP-myc overexpression completely abolished colony formation. These results indicate that OMD and PRELP suppression might be important for the transition from normal epithelial cells to mesenchymal-like cancer cells. Additionally, we tested the cell growth in a 3D environment using Matrigel to investigate growth under partial anchorage conditions. Control EJ28 cells grew well and showed a “spread-like” morphology (Figure 3f), as observed in standard cell culture dishes. However, OMD or PRELP overexpressing cells tended to make cell aggregates, suggesting that OMD and PRELP may influence cell migration. To address this, we performed the Boyden chamber assay with Matrigel-coated transwells. The assay clearly demonstrated that the overexpression of OMD or PRELP strongly suppressed cell migration and invasion (Figure 3g,h). The effect of OMD and PRELP overexpression on cell migration was also tested in standard 2D conditions with the scratch wound assay, where a small inhibition of the wound recovery was observed (Figure S4g). Collectively, these results suggest that the two proteins affect colony formation, migration, and invasion capabilities of cancer cells in a substrate-dependent manner.

As OMD and PRELP are secreted proteins, to confirm that the observed effects are mediated by the extracellular forms, we performed a co-culture assay, in which EJ28 cells (Cell A) overexpressing OMD or PRELP were cultured in the chamber above tester EJ28 cells (Cell B) (Figure 3i). OMD and PRELP significantly suppressed the growth of the lower layer of EJ28 tester cells (Figure 3j), as we expected.

### 2.4. The Relation between OMD or PRELP and Tight Junction Formation

**Figure 4:**
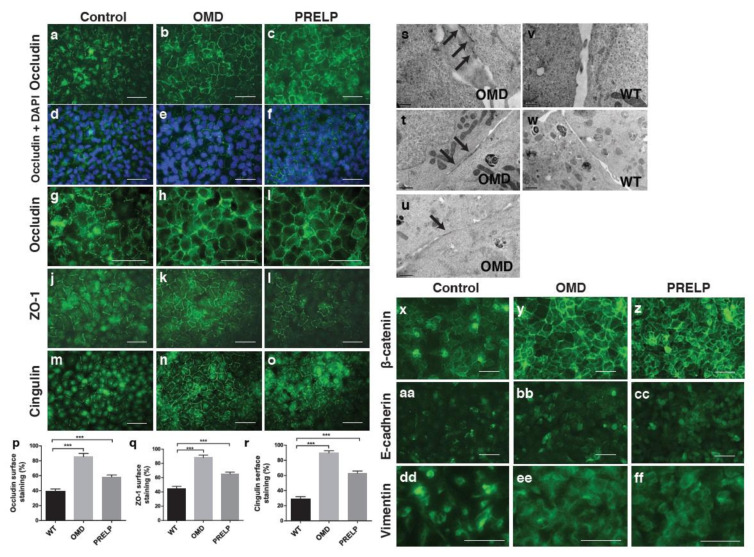
Effect of OMD or PRELP overexpression on tight junction in EJ28 cells. (**a**–**i**) Occludin antibody staining of OMD or PRELP expressing EJ28 cells; low magnification (**a**–**c**), overlaid with DAPI (**d**–**f**), enlarged (**g**–**i**). **(j**–**l**) ZO-1 staining. (**m**–**o**) Cingulin staining. Scale bar represents 100 μm (**a**–**o**). (**p**) Quantification of occluding staining. (**q**) Quantification of ZO-1 staining. (**r**) Quantification of cingulin staining. (**s**–**w**) Electron microscope (EM) analysis of cell-cell junction. OMD expressing EJ28 cells (**s**–**u**) and wild type (WT) EJ28 cells (**v**,**w**). Tight junctions are indicated by arrows. Scale bar represents 1 μm (**t**,**u**,**w**) and 0.5 μm (**s**,**v**). OMD overexpression strongly activates tight junction formation. (**x**–**ff**) Antibody staining of confluent monolayer; β-catenin (**x**–**z**), E-cadherin (**aa**–**cc**), and vimentin (**dd**–**ff**). Scale bar represents 100 μm (**x**–**ff**). *** indicates *p* < 0.001.

We examined the status of tight junctions of EJ28 cells using antibodies against occludin (Figure 4a–i), ZO-1 (Figure 4j–l), and cingulin (Figure 4m–o). In confluent monolayers of the control EJ28 cells, we observed partial staining at cell–cell interfaces, covering around 40% of the total cell–cell surface for occludin, (40% of total cell–cell surface), ZO-1 (46%), and cingulin (30%) (Figure 4p–r). This appearance of partial junction staining is found in cancer cell lines (personal communication, Karl Matter). Interestingly, the overexpression of OMD resulted in enhanced and continuous junctional staining of all three markers, covering almost the whole cell periphery (Figure 4b,h,k,n). PRELP overexpression had a similar effect, where tight junction formation was also markedly increased compared to the control cells. This enhanced junctional staining was accompanied by a reduction of the cytoplasmic staining of the corresponding markers. To further confirm the formation of tight junctions, the control EJ28 cells and OMD overexpressing cells were examined by electron microscopy. A large number of tight junctions were observed in OMD overexpressing cells (Figure 4s–u). However, we failed to detect any tight junctions in the control EJ28 cells (Figure 4v,w).

Subsequently, to determine the effect on adherens junctions, we examined the expression of β-catenin, E-cadherin, and vimentin. Figure 4x shows that in the control group, many cells have a weak β-catenin localization in the nuclei. On the other hand, in OMD or PRELP overexpressing cells, β-catenin was almost exclusively localized at the plasma membrane, and the strength of the staining was much higher than the control (Figure 4x–z). E-cadherin staining was slightly enhanced (Figure 4aa–cc), indicating that OMD and PRELP activate adherens junctions. To test how OMD and PRELP regulated cell–cell adhesion, we examined the expression of vimentin, an EMT marker. The major characteristics of epithelial cells are cell polarity, strong cell–cell integrity, and anchorage-dependent growth. Cancer initiation in epithelia is always associated with EMT [25,26]. After conversion to mesenchymal cells, these cells can grow in an anchorage-independent manner, as observed in almost all cancer cells. Figure 4dd–ff shows that vimentin was more localized around or in the nucleus, while OMD or PRELP-expressing cells showed a diffuse expression of vimentin in the cytosol. This suggests that OMD and PRELP may regulate cell–cell adhesion through EMT.

### 2.5. Signal Pathways Regulated by OMD and PRELP

**Figure 5:**
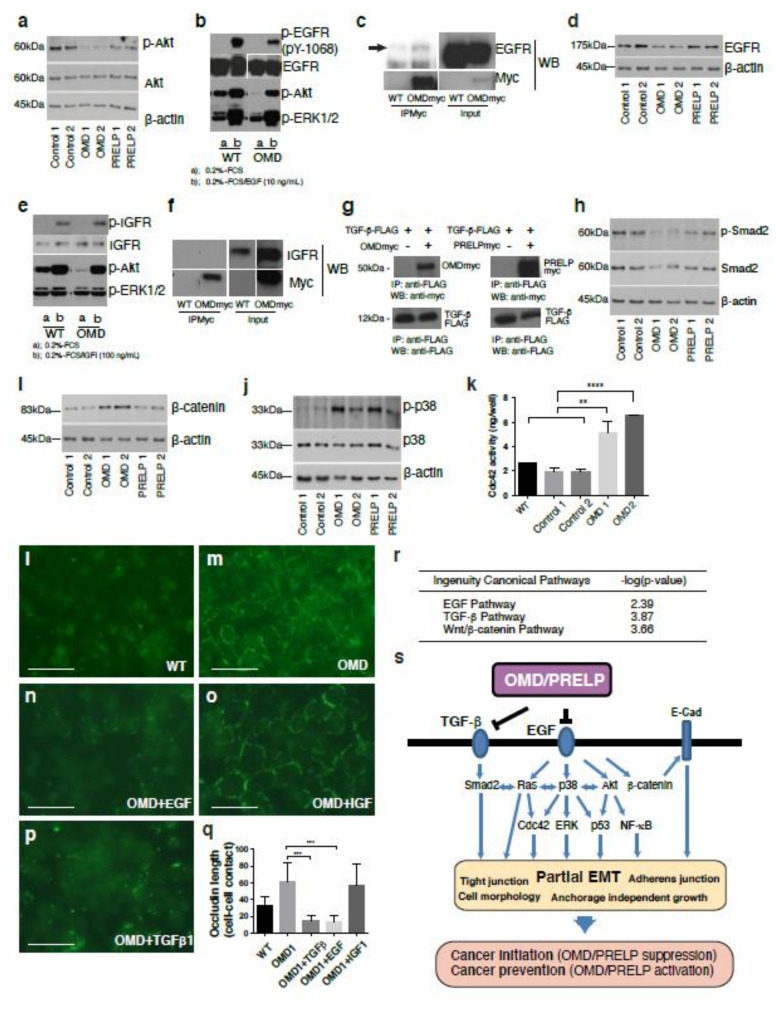
Mechanism of OMD or PRELP-mediated regulation of tight junction. Various effects of OMD and PRELP were examined in vitro using OMD or PRELP stably overexpressing EJ28 bladder cancer cell lines. OMD1 and OMD2 indicate different stable clones. (**a**) Effect of OMD or PRELP overexpression on Akt phosphorylation. (**b**) Effects of OMD overexpression and EGF application on EGF receptor, Akt, and ERK phosphorylation. (**c**) Interaction between OMD and EGF receptor. (**d**) Effect of OMD or PRELP on the total amount of EGF receptor. (**e**) Effects of OMD overexpression and IGF-1 application on phosphorylation of the IGF receptor, Akt, and ERK. (**f**) Interaction between the OMD and IGF receptors. (**g**) Binding of OMD or PRELP with TGF-β. (**h**) Effect of OMD or PRELP on Smad2 phosphorylation. (**i**) Effect of OMD and PRELP on the total levels of β-catenin protein expression. (**j**) Effect of OMD or PRELP on phosphorylation of p38. All original Western blotting data are shown in Figure S8. (**k**) Effect of OMD on cdc42 activity. (**l**–**p**) Effect of EGF, IGF-1, and TGF-β 1 application on tight junction formation of confluent OMD overexpressing EJ28 cell monolayers. Occludin staining of normal EJ28 cells (**l**) and OMD expressing EJ28 cells (**m**). Effect of 10 ng/mL EGF (**n**), 100 ng/mL IGF-1 (**o**), or 10 ng/mL TGF-β 1 (**p**) on occludin staining of EJ28 cells overexpressing OMD. Scale bar represents 100 μm. (**q**) Quantification of occludin-positive cell–cell junctions. (**r**) TGF-β, EGF, and Wnt pathways are affected in OMD^−/−^ mouse bladder. Ontological analysis of the expression profiling data obtained in Figure 8. (**s**) Schematic model of OMD/PRELP function. The uncropped Western Blot figure in Supplementary Figure S8. **, **** indicate *p* < 0.01, *p* < 0.0001, respectively.

The gene expression profiling experiment revealed that OMD and PRELP were involved in the regulation of various components of several ligand-induced signaling pathways, including the IGF-1, Wnt, EGF, and TGF-β pathways. We aimed to determine the molecular mechanisms of OMD/PRELP activity using EJ28 stable cell lines that overexpress the two proteins. In the expression profiling data (Figure 2d), the Akt level was significantly affected by both OMD and PRELP. We found that OMD and PRELP overexpression downregulated the phosphorylation of Akt (Figure 5a), and OMD overexpression downregulated the phosphorylation of ERK1/2 (Figure 5b). Akt phosphorylation is known to be regulated by the EGF and IGF pathways [27,28]. Figure 5a and b show that upon EGF treatment (10 ng/mL), Akt phosphorylation was decreased in the OMD overexpressing cells compared to the control. EGF induced the phosphorylation of tyrosine-1068 of the EGFR, and this phosphorylation was suppressed by OMD expression (Figure 5b). ERK1/2 phosphorylation was elevated by exogenous EGF, and this phosphorylation was also suppressed by OMD (Figure 5b). Co-immunoprecipitation assays revealed that OMD was bound to the EGFR (Figure 5c). Total EGFR protein was reduced in OMD transfected cells (Figure 5d). Inhibition of the EGF pathway is known to lead β-catenin localization to the cell membrane [29], which we observed in OMD/PRELP activation (Figure 4x).

IGF activated Akt through the phosphorylation of the IGF-1R; however, OMD overexpression did not inhibit the IGF-mediated phosphorylation of Akt (Figure 5e) in our assays. In addition, we did not detect any direct interaction of OMD with the IGF receptor (Figure 5f). All the SLRP family members previously studied directly interact with TGF-β family members and regulate transcription of their targets via the phosphorylation of Smad2 [2]. Indeed, OMD and PRELP directly bound to TGF-β protein (Figure 5g) and resulted in Smad2 phosphorylation suppression, particularly in OMD (Figure 5h). The effect of OMD and PRELP on EGFR, β-catenin, and Smad2 were quantitated and the results are shown in Figure S5.

We found that OMD overexpression significantly increased the total amount of β-catenin (Figure 5i). However, we could not detect a change of Wnt-mediated transcription activity by the TOPFLASH assay (unpublished data). Taken together with our finding that OMD causes the translocation of β-catenin to the plasma membrane (Figure 4x–z), this suggests that the increased β-catenin mainly contributes to its adherens junction-related function.

The downstream segments of ligand-induced signaling pathways are remarkably interconnected with each other in context-dependent manners. Thus, we examined two common downstream components of the EGF and TGF-β pathways, p38 and cdc42, as the OMD or PRELP mediated in vitro phenotypes reported in this paper are similar to those caused by p38 or cdc42 modulation [30,31,32]. Moreover, our expression profiling analysis indicated the importance of the cdc42 and p38 pathways in this context (Figure 2d). We found that OMD and PRELP overexpression increased the phosphorylation of p38 (Figure 5j), and OMD activated cdc42 (Figure 5k).

Finally, we examined the contribution of OMD-mediated inhibition of pathways to the regulation of tight junctions. TGF-β, IGF, and EGF pathways are well known as major pathways to regulate EMT and mesenchymal–epithelial transition (MET). OMD overexpressing EJ28 cells were treated with either EGF (10 ng/mL), TGF-β (10 ng/mL), or IGF-1 (100 ng/mL) protein, and their effects on tight junction formation were assessed. Cellular response was confirmed by analysis of phosphorylation of ERK1/2, AKT, and Smad2. Figure 5l–q shows that EGF and TGF-β strongly inhibited OMD-induced tight junction formation, while IGF-1 had no effect, suggesting that the OMD-mediated regulation of both EGF and TGF-β pathways is important for the regulation of tight junctions. In addition, OMD overexpression induced the translocation of β-catenin to the plasma membrane (Figure 4x), which was accompanied by an increase in the total expression levels of β-catenin (Figure 5i). Such effects were previously reported as phenotypes caused by EGF pathway inhibition [29]. Later, we will show another gene expression profiling using bladder tissues isolated from OMD^−/−^ or PRELP^−/−^ mice (Figure 8). The ontological analysis shows that indeed, OMD/PRELP regulate EGF and TGF-β pathways (Figure 5r)

Our results demonstrate that the OMD-mediated simultaneous regulation of TGF-β and EGF pathways is important for the maintenance of cell–cell adhesion (Figure 5s).

### 2.6. Tumor Progression in a Mouse Xenograft Model

**Figure 6:**
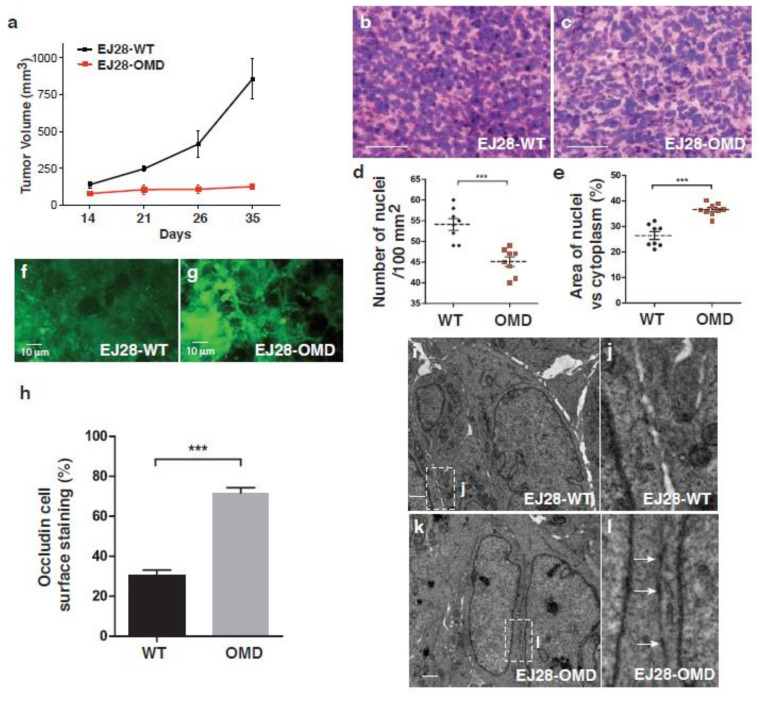
Mouse xenograft model overexpression of OMD. (**a**) Xenograft of EJ28 cells stably expressing OMD. Tumor volume progression graph. EJ28-*WT* (*n* = 5) and EJ28-OMD (*n* = 5). (**b**–**e**) Histology of xenografted tissues; H&E staining of control EJ28 cells (**b**) and EJ28 cells overexpressing OMD (**c**), comparison of the number of nuclei in 100 µm^2^ of sections (**d**), comparison of the ratio of nucleus vs cytosol. (**e**) Scale bar represents 100 μm (**b**,**c**). (**f**,**g**) Occludin staining of control EJ28 tumor (**f**) and OMD overexpressing EJ28 tumor (**g**). (**h**) Quantification of occludin staining. The stained percentage of cell surfaces was measured. (**i**) EM of control EJ28 cells. (**j**) Enlarged from (**i**). (k) EM of OMD-overexpressing EJ28 tumor cells. Scale bar represents 1 μm (**i,k**). (l) Enlarged from (**k**). Tight junctions are shown with arrows. *** indicates *p* < 0.001.

In order to examine the in vivo effects of OMD overexpression in cancer development, we performed mouse xenograft experiments using stably transformed EJ28 cells. When EJ28 cells were grafted in nude mice, the control EJ28 cancer cells grew well, while OMD-expressing EJ28 cells did not grow at all (Figure 6a). These observations are in accordance with the decreased anchorage-independent growth we observed in vitro (Figure 3d–e). Haemotoxylin and Eosin (H&E) staining of tumor sections showed that the density of nuclei was reduced and the nuclear–cytoplasmic ratio was increased in OMD-overexpressing samples (Figure 6b–e). Moreover, occludin staining revealed that OMD-expressing EJ28 cells have a more organized structure and stronger tight junctions (Figure 6f–h). Next, we analyzed the ultrastructure of the xenografted cells by electron microscopy. This analysis showed that adjacent cells of the control samples intercellular spaces between neighboring cells are always visible, and almost no tight junctions can be observed (Figure 6i,j), while the OMD-expressing xenografts are in close contact and form multiple tight junctions (Figure 6k,l). These results confirmed that OMD/PRELP overexpression enhances cell–cell adhesion and suppresses cancer development in vivo.

### 2.7. OMD^−/−^ or PRELP^−/−^ Mice and Tight Junctions between Umbrella Cells

Next, we established constitutive *OMD*^−/−^, *PRELP*^−/−^, and *OMD*^−/−^/*PRELP*^−/−^ double knockout mice (Figure S6a–d). The knockouts were designed to target exons 2 and 3, resulting in the complete removal of protein coding sequences while knocking in the β-galactosidase gene under the *OMD* and *PRELP* promoters, respectively. The mice were viable and fertile, and no severe developmental defects were observed. *OMD* and *PRELP* expression in mice were analyzed by qRT-PCR (Figure S6e,f).

*OMD* and *PRELP* were expressed in all organs tested in various levels (Figure S6g,h for mouse, Figure S6i,j for human). To characterize the expression in the bladder, we assayed β-galactosidase activity in heterozygous *OMD*^+/−(LacZ)^ and *PRELP*^+/−(LacZ)^ mice. We observed β-gal-positive cells only in the epithelial layer (Figure S6k,l). A similar pattern was found by the in situ hybridization with the *OMD* or *PRELP* gene probe (Figure S6m,n). The bladder epithelium contains three cell types: basal cells, intermediate cells, and superficial umbrella cells [33]. To identify which cell types express OMD or PRELP, bladder sections were co-stained with β-gal and uroplakin-III (umbrella), CK18 (umbrella), CK5 (basal), or laminin (basement membrane of epithelium) antibody. In *OMD*^+/−^ mice, β-gal positive cells were always co-localized with a subpopulation of the uroplakin-III and CK18 positive cells, but not with CK5 or laminin (Figure S6o–r). We also stained with Ki67 (proliferative) markers (Figure S6s), but there was no overlap staining. PRELP showed an expression pattern similar to that of OMD (Figure S6t–x). These results indicate that at any one time, the active transcription of *OMD* and *PRELP* is occurring in a subpopulation of umbrella cells.

**Figure 7:**
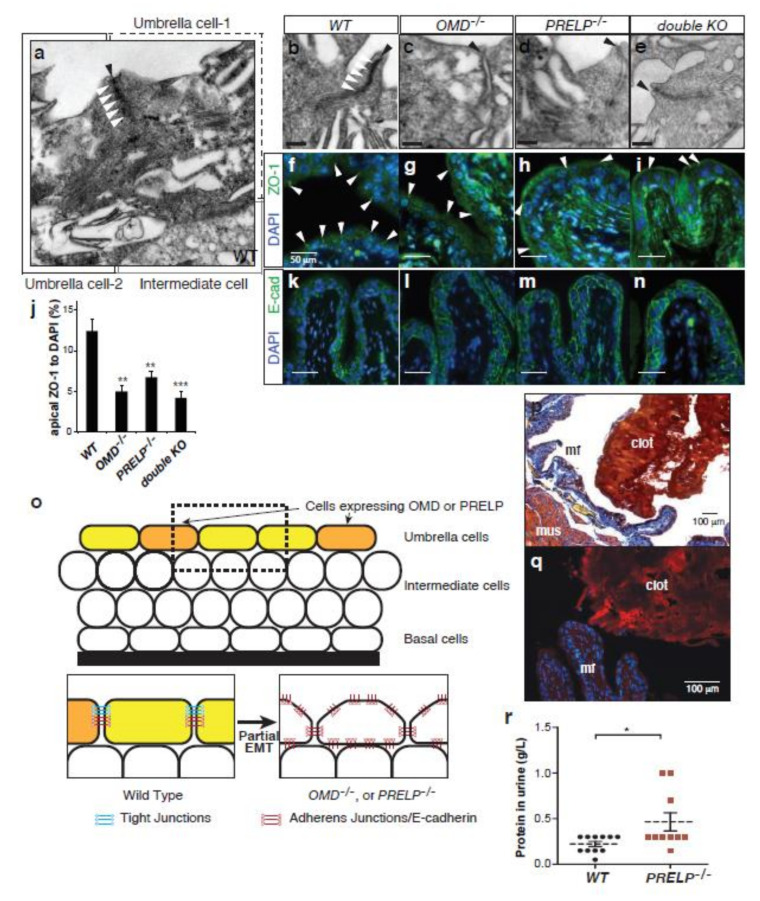
*OMD* or *PRELP* knockout resulted in a loss of tight junctions between bladder umbrella cells. (**a**–**e**) EM analysis of *WT* (**a,b**), *OMD*^−/−^ (**c**), *PRELP*^−/−^ (**d**), and their double (**e**) knockout bladders at 3 months old. A wide view of *WT* bladder epithelia, which includes two umbrella cells and an intermediate cell. The apical side of the cell–cell surface of umbrella cells (black arrowhead) are strongly sealed by dense tight junctions (white arrowheads) (**a**). Apical side of umbrella cell–cell interfaces. Black arrowheads; cell–cell interfaces. White arrowheads; tight junctions (**b**–**e**). Scale bar represents 200 nm. (**f**–**j**) Analysis of ZO-1 staining of a 3-month-old bladder. ZO-1 staining between umbrella cells is indicated by white arrowheads (**f**). Quantification of ZO-1 staining (**j**). (**k**–**n**) E-cadherin staining of a 3-month-old bladder. (**o**) Model of cell–cell adhesion in bladder epithelial cells. (**p**) Phosphotungstic acid hematoxylin (PATH) staining of 3-month-old *PRELP*^−/−^ bladder. PATH staining stains fibrin and erythrocytes. (**q**) Fibrin antibody staining of 3-month-old *PRELP*^−/−^ bladder. (**r**) Analysis of urinary fibrin. Urine samples were collected from *WT* and *PRELP*^−/−^ mice at the morning and were tested using Multistix (SIEMENS). *, **, *** indicate *p* < 0.05, *p* < 0.01, *p* < 0.001, respectively.

Umbrella cells are connected to each other strongly by tight and adherens junctions [33]. We examined the effect of OMD or PRELP deficiency on umbrella cell junctions. Electron microscopy images indicated that the apical–lateral interfaces between *WT* bladder umbrella cells were tightly sealed by dense tight junctions (Figure 7a,b). However, strong tight junctions were markedly reduced in OMD^−/−^, PRELP^−/−^, or the double knockout mice (Figure 7c–e). The reduction at the lateral surface was confirmed by immunostaining with the tight junction marker ZO-1. In the *WT*, ZO-1 staining was located at the lateral cell surface (Figure 7f). In *OMD***^−/−^** or *PRELP*^−/−^ bladder tissues, the ZO-1 signal at the lateral cell surface was significantly reduced (Figure 7g–j). Adherens junctions are localized in the lateral cell–cell surface between umbrella cells, below the tight junction level. In *WT* mice, the adherens junctions were visible in the basolateral surface of umbrella cells, as marked by E-cadherin staining (Figure 7k), while in *OMD*^−/−^, *PRELP*^−/−^, and the double knockout mice, E-cadherin was localized in the whole cell surface (Figure 7l–n). This demonstrates that the disruption of tight junctions enables E-cadherin to migrate to the apical side of the cell membrane. These observations indicate that OMD or PRELP depletion results in the induction of a partial EMT state, which is characterized by the loss of tight junctions but not adherens junctions (Figure 7o).

One of the major functions of tight junctions in the bladder is to form the blood–urine barrier to block the leakage of fluids into the bladder [34]. In accordance with this function, deletion of the *PRELP* gene resulted in the formation of clots containing fibrin/fibrinogen in the bladder lumen (Figure 7p,q) and the leakage of proteins into the urine (Figure 7r).

### 2.8. Expression Profiling of OMD^−/−^, PRELP^−/−^ Bladder Epithelia

**Figure 8:**
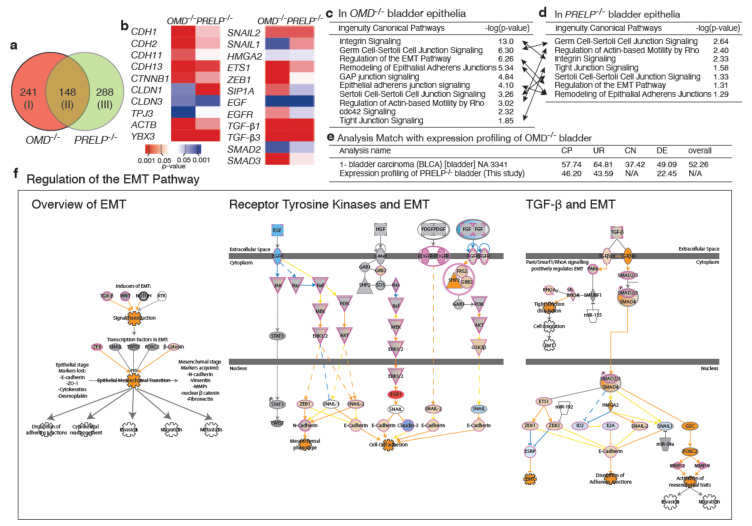
Expression profiling of *OMD*^−/−^, *PRELP*^−/−^ mouse bladder epithelia. Expression profiling was performed using *OMD*^−/−^, *PRELP*^−/−^ mouse bladder epithelia. Data were analyzed, and the following figures were made through the use of IPA (QIAGEN Inc., https://www.qiagenbioinformatics.com/products/ingenuitypathway-analysis). (**a**) Significantly affected gene numbers, including both up and downregulated. (**b**) Expression of genes related to cell–cell adhesion and EMT. (**c,d**) Significantly affected cell adhesion-related pathways in *OMD*^−/−^ (**c**) and *PRELP*^−/−^ (**d**). The same pathways are connected by arrows. (**e**) Similarity of expression profiling data. Using Analysis Match software (Ingenuity Pathway Analysis, IPA, Qiagen), we examined the similarity of expression profiling data of OMD^−/−^ retina with the already deposited publicly available expression profiling dataset and those of PRELP^−/−^ retina. OMD^−/−^ retina data showed high similarity with PRELP^−/−^ retina. The public database search revealed that bladder cancer-related datasets showed high similarity in all categories. CP; canonical pathways, UR; upstream regulators, CN; causal networks, DE; downstream effectors. (**f**) Schematic drawing of “Regulation of the EMT pathway” in *OMD*^−/−^ vs. *WT*. Drawing was slightly modified from the original of “Regulation of the EMT Pathway”. This image was created by Ingenuity Pathway Analysis according to their rule.

To consolidate our hypothesis that OMD and PRELP contribute to the maintenance of cell–cell adhesion and the inhibition of EMT, we performed gene expression profiling by RNA-seq using isolated bladder epithelia from *WT* mice (*n* = 3), *OMD*^−/−^ (*n* = 5), and *PRELP*^−/−^ (*n* = 3). Similarly to our previous gene expression analysis data (Figure 2), 148 genes were commonly affected both in *OMD*^−/−^ and in *PRELP*^−/−^ (Figure 8a), indicating their partial functional redundancy.

These genes include components of cell–cell adhesion and EMT (Figure 8b). Ontological analysis using the IPA showed that EMT-related events such as “Regulation of the Epithelial–Mesenchymal Transition Pathway” (*OMD*^−/−^, *z* = 6.26, *PRELP*^−/−^, *z* = 1.31) (Figure 8c–e) were significantly affected in both *OMD*^−/−^ and *PRELP*^−/−^ bladder epithelia. Additionally, cell–cell adhesion-related pathways, which is a consequence of EMT, such as “Tight Junction Signaling”, and “Germ Cell–Sertoli Cell Junction Signaling”, were significantly affected both in *OMD*^−/−^ and *PRELP*^−/−^, confirming their involvement in the maintenance of the epithelial junctional barrier.

The ontological analysis also revealed that many cancer-related pathways are more strongly affected (Figure S7a,b), even to a higher extend compared to the gene expression profiling performed in cell lines (Figure 2). Many oncogenes and tumor-suppressor genes are strongly affected (Figure S7c). Figure S6d shows the schematic diagram of “Molecular Mechanisms of Cancer” pathway, affected in *OMD*^−/−^ (*z* = 15.2), indicating that a majority of cancer-related regulators such as NF-kB, p53, myc, Ras, c-Jun/c-Fos, TGF-β R1/2, and RB are significantly affected. Since the host mouse strain C57BL/6J is not known to hold tumorigenic mutations in the above proteins, these data confirm that in parallel with their ability to regulate EMT and cell–cell integrity, OMD and PRELP have the ability to influence cancer-related activities. Furthermore, in order to know how deeply the OMD suppression in bladder cancer contributes to the properties of bladder cancer, we searched already deposited publicly available expression profiling datasets that showed similarities with that of OMD^−/−^ retina. This analysis revealed that many cancer-related public datasets showed strong similarity with our OMD^−/−^ dataset. In particular, as shown in Figure 8e, both bladder transitional cell carcinoma and bladder carcinoma showed the strong similarity [35,36]. This result demonstrates the significant contribution of OMD suppression in human bladder cancer initiation and/or progression. In addition, we examined the similarity between the OMD^−/−^ and PRELP^−/−^ expression profiling datasets using the Analysis Match software. Figure 8e shows the high similarity between OMD^−/−^ and PRELP^−/−^, supporting the results in Figure 8a–d.

### 2.9. Breakdown of the Umbrella Cell Layer in OMD^−/−^ and PRELP^−/−^ Mice

**Figure 9:**
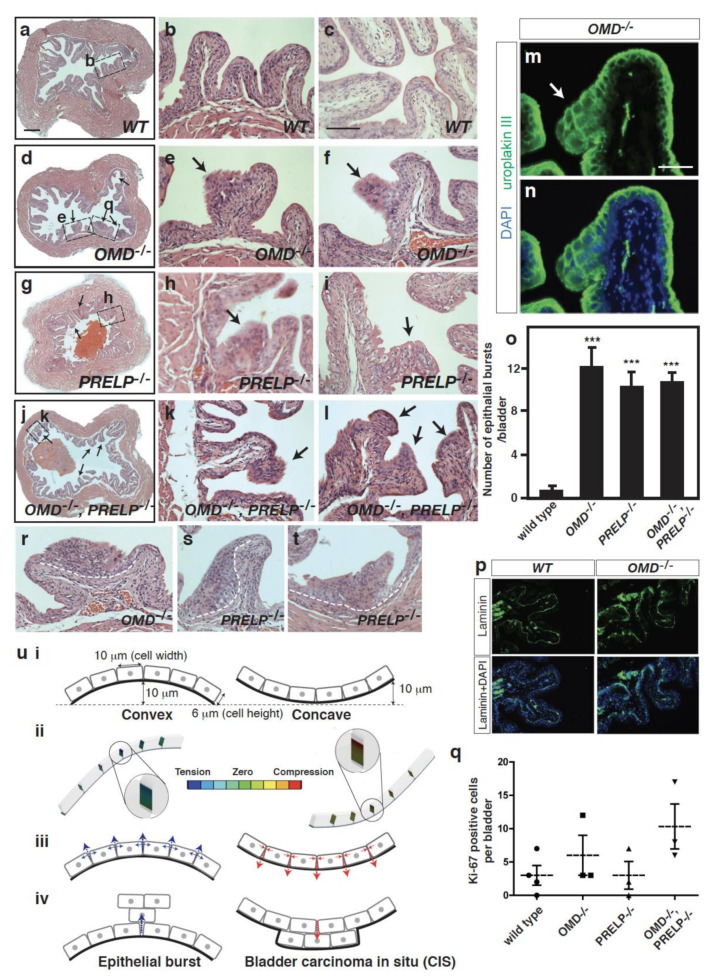
*OMD*^−/−^, *PRELP*^−/−^, and their double knockout mice spontaneously initiate bladder papillary cancer. (**a**–**l**) 3-month-old bladder of *WT* (**a**–**c**), *OMD*^−/−^ (**d**–**f**), *PRELP*^−/−^ (**g**–**i**), and their double knockout (**j**–**l**). A low magnification and two high magnification images are shown in order. Epithelial bursts are indicated as arrows. Some enlarged areas are indicated as boxes in low-magnification images. Scale bar represents 500 μm (**a**,**d**,**g**,**j**) and 100 μm (**b**,**c**,**e**,**f**,**h**,**i**,**k**,**l**). (**m**–**o**) Uroplakin III antibody staining of an epithelial burst of OMD^−/−^. Uroplakin staining (m), overlaid view of uroplakin III and DAPI (**n**). Scale bar represents 50 μm (**m**,**n**). Quantification of epithelial burst number per bladder; *WT* (*n* = 7), *OMD*^−/−^ (*n* = 7), *PRELP*^−/−^ (*n* = 6), *OMD*^−/−^, *PRELP*^−/−^ (*n* = 3) (**o**). In quantification, we examined six to seven 10 µm slices from each bladder. Each slice was separated around 200 µm in the bladder, and these slices covered the whole bladder except their edges. (**p**) Laminin antibody staining of *WT* and *OMD*^−/−^ bladders. (**q**) Ki-67 staining positive cells in bladder. (**r**–**t**) Carcinoma in situ (CIS)-like structures in *OMD*^−/−^ (**r**) and *PRELP*^−/−^ (**s**,**t**). (**u**) Computational models for the mouse and human epithelial dysplasia. Conditions of models (**u-i**). Calculated forces between cells (**u-ii**). Direction of dysplasia (**u-iii**). Epithelial burst-like dysplasia and carcinoma in situ-like dysplasia (**u-iv**). *** indicates *p* < 0.001.

We made 10 µm paraffin section series from whole bladder specimens of *WT*, *OMD*^−/−^, *PRELP*^−/−^, and double knockout mice and examined the fine structure of the urothelium. In the *WT* mice, bladder umbrella cells form a clear single epithelial layer at the apical side of the urothelium and function as a barrier to the toxic bladder fluid (Figure 9a–c). In contrast, all of the bladder tissue samples from *OMD*^−/−^, *PRELP*^−/−^, and the double knockout mice showed points of breakdown/dysplasia of the urothelium (Figure 9d–l). We here termed these histological structures as “epithelial bursts”. Furthermore, histological observation and bladder marker staining showed that the spread cells of the epithelial bursts originated from umbrella cells expressing uroplakin-III (Figure 9m,n), while their number was significantly increased in *OMD*^−/−^ or *PRELP*^−/−^ mice (Figure 9o). Of note, no obvious abnormalities were seen in the basal and intermediate cell layers (Figure 9p). To investigate whether the epithelial bursts are associated with aberrant cell proliferation, we performed immunohistochemical analysis using the Ki67 proliferation marker. There are few Ki67-positive cells in the *WT* bladder urothelium, and their number is only slightly increased in the *OMD*^−/−^ and the double knockout samples, suggesting that the epithelial bursts do not result from increased proliferation (Figure 9q).

In humans, carcinoma in situ (CIS) appears histologically as a flat dysplasia of umbrella cells and is recognized as an early sign of malignant bladder cancer. However, an epithelial burst-type dysplasia, as seen in the *OMD*^−/−^ and *PRELP*^−/−^ mouse bladders, has not been recognized. The luminal mouse bladder is consistently covered by convex mucosal folds, while the human bladder surface is relatively flat or slightly concave. During our histological analysis, we observed a simple flat dysplasia of umbrella cells in the concave areas of mouse bladder as in human CIS (Figure 9r–t), suggesting that the structural difference of dysplasia might result from the different urothelium structure: convex vs. concave. To address this, we developed a mathematical simulation to visualize the direction of epithelial layer breakdown through the calculation of the forces created on convex and concave structures (Figure 9u). The model demonstrated that in a convex structure, the basal side of the epithelial layer was sealed, and the epithelial cells tended to escape to the apical side, similar to an epithelial burst. On the other hand, in a concave structure, the apical side was sealed, and the dysplasia cells tended to move under the epithelial layer. Supporting our analysis, Messal et al. has recently reported that a mechanical tension model for tissue curvature can instruct the direction of cancer morphogenesis [37]. These model-based analyses suggest that OMD and/or PRELP deletion can result in a defect in maintenance of the umbrella cell layer, as observed in human bladder CIS.

### 2.10. Some PRELP^−/−^ Mice Spontaneously Initiate Bladder Papillary Cancer

**Figure 10:**
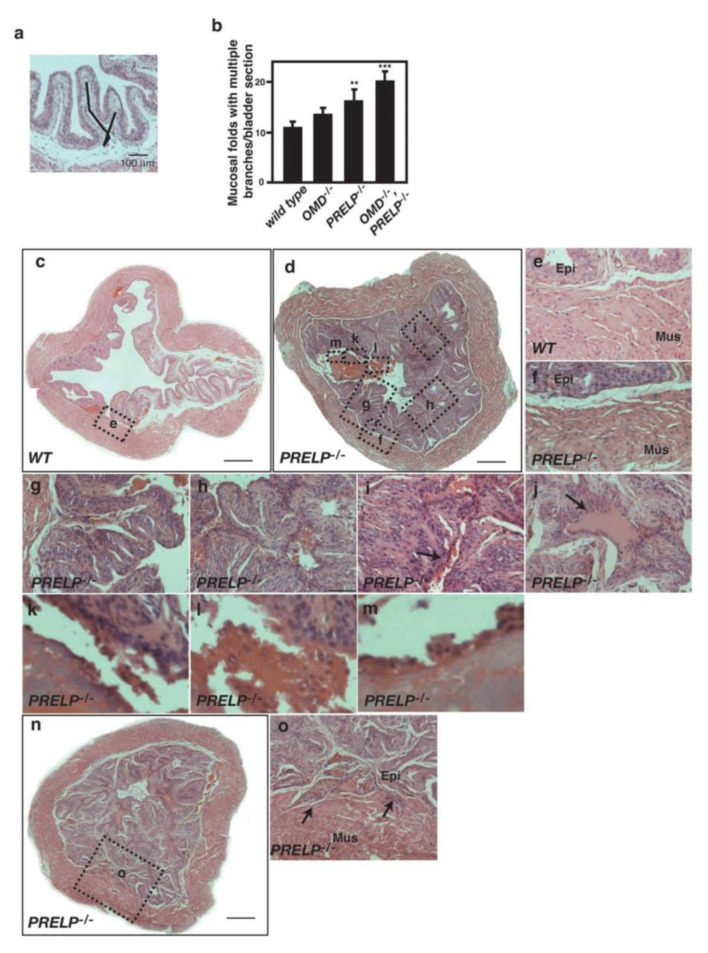
*PRELP* knockout mice spontaneously initiate early stages of bladder cancer. (**a**) H&E-stained section showing a branched mucosal fold. (**b**) Number of mucosal folds with multiple branches. In each bladder, we have examined two sections in the medial region of bladder. (**c**) H&E-stained section of a *WT* mouse bladder at 3 months of age. (**d**) Bladder papillary cancer in *PRELP*^−/−^ at 3 months. Scale bar represents 500 μm (**c,d**). The bladder lumen is almost completely filled by mucosal folds with multiple branches and fused mucosal folds. Clots formation is observed. Enlarged regions in the following panels are indicated by the dotted boxes. (**e**) *WT* bladder muscularis (Mus) and epithelial tissue (Epi). (**f**) *PRELP*^−/−^ bladder muscularis. (**g**) Mucosal fold with multiple branches. (**h**) Partially fused mucosal folds with multiple branches. (**i**) Fused mucosal folds. The arrow points secretion of materials to lumen. (**j**) Fused mucosal folds. Deposited material is enriched in fused folds (arrow). (**k**) Separation of epithelial cells with sticky material. (**l**) Aggregation of separated cells with clot materials. (**m**) The clot is covered by a layer of cells. (**n**) T1 stage bladder cancer in *PRELP*^−/−^ at 3 months. (**o**) Epithelial papillary cancer integration into muscularis (arrows). ** and *** indicate *p* < 0.005, and *p* < 0.001, respectively.

On analysis of bladders from *OMD*^−/−^, *PRELP*^−/−^, and the double knockout mice, we found that *OMD*^−/−^, *PRELP*^−/−^, and double KO bladders showed a slightly increased number of mucosal folds with multiple branches (Figure 10a,b). Interestingly, in one-third of the *PRELP***^−/−^** and double knockout mice but not in OMD^−/−^ mice, the bladder developed abnormal urothelia with hyperplasia, resulting in a pattern of papillary growth on a normal muscularis (Figure 10c,e in WT, d, f–o in PRELP). This phenotype seen in some *PRELP*^−/−^ bladders is similar to some types of human bladder papillary cancer (https://www.proteinatlas.org/learn/dictionary/pathology/urothelial+cancer).

We observed various stages of papillary cancer progression such as mucosal folds with multiple branches (Figure 10g), partially fused mucosal folds (Figure 10h), and completely fused mucosal folds (Figure 10i,j). The process of clot formation was also observed, including small aggregates of proteinaceous material secreted from umbrella cells (Figure 10i), larger aggregates in which clumps of cells were embedded (Figure 10k,l), and large acellular clots covered with a single layer of cells (Figure 10m). We observed early signs of cancer invasions into the underlying muscularis (Figure 10n,o).

## 3. Discussion

### 3.1. ECM Proteins and Cancer Initiation

OMD and PRELP are secreted ECM proteins, belonging to the Class II SLRP subfamily [38,39,40]. SLRP family members were originally identified as abundant proteins within the ECM of cartilage, connecting tissues and differentiating osteoblasts [41,42,43]. ECM proteins of the tumor microenvironment play important roles in many aspects of cancer initiation and progression [44]. One member of the SLRP family, decorin expression, decreases on the malignant transformation of tumor cells. Thirty percent (30%) of decorin knockout mice developed spontaneous intestinal tumors [13]. On the other hand, in an inflammation murine model, decorin is upregulated in endothelial cells and facilitates the downregulation of tight junctions [45]. This suggests that inflammation may affect OMD and PRELP function.

Here, we have demonstrated that OMD and PRELP function to maintain epithelial cell–cell integrity in urothelial cells through the inhibition of partial EMT. At epithelial cancer initiation, EMT is required, while MET is observed at cancer metastasis. Recent comprehensive expression profiling analyses in bladder and other epithelial cancers have revealed a novel concept of partial EMT [46,47,48,49]. The typical partial EMT state is the loss of tight junctions without affecting adherens junctions [48]. This is particularly important for understanding cancer initiation. In bladder cancer, a loss of E-cadherin expression is used as a marker of advanced bladder cancer, suggesting that the partial EMT state might be associated with early-stage bladder cancer. The tight junctions between umbrella cells in *OMD*^−/−^ and *PRELP*^−/−^ mice disappeared, while adherens junctions were maintained, indicating a typical partial EMT state. The loss of tight junctions resulted in disruption of the apical–basal polarity of umbrella cells, which is demonstrated by uniform E-cadherin staining around umbrella cells. Moreover, the partial EMT state we observed is susceptible for breakdown of the umbrella-cell layer, which might be related to cancer initiation. Collectively, our findings might be the first demonstration of partial EMT state and associated bladder cancer initiation in mice.

### 3.2. OMD and PRELP and NMIBC Initiation

*OMD*^−/−^ or *PRELP*^−/−^ mice showed many breakdown sites in the umbrella-cell layer, and one-third of *PRELP*^−/−^ developed large-scale papillary cancer without muscle invasion. A large region of chromosome 9q, including the *OMD* gene, is deleted in half of NMIBC cases [50]. The deletion is associated with the initiation of NMIBC [51]. *PTCH* and *TSC1* were proposed to be the critical tumor-suppressor genes in 9q deletions [52,53], but this hypothesis is controversial [54]. Rather, with the present study, we propose *OMD* as a novel 9q-residing tumor-suppressor gene involved in cancer bladder initiation.

NMIBC is clinically classified as Ta, T1, or CIS. CIS is proposed to originate from umbrella cells because the cells in CIS are positive to umbrella cell markers such as CK20 [55]. Recent comprehensive expression profiling analysis classified NMIBC into three classes. Among these, Class 2 has the expression of CIS type markers, and Class 2 is defined based on the expression of EMT marker genes [20]. *OMD*^−/−^ or *PRELP*^−/−^ showed two types of breakdown of the umbrella layer: epithelial bursts and CIS-like structures. Our mathematical model indicates that the difference between the two breakdowns reflects the structural differences of the epithelia. We propose that umbrella-layer breakdown mediated by the loss of OMD and PRELP may initiate CIS. Some bladder cancers are thought to originate from the umbrella cells [51], because selective overexpression of a mutant H-Ras in umbrella cells resulted in low-grade papillary tumors [56,57,58].

Additionally, *PRELP*^−/−^ mice tended to form protein clots, including fibrin, in the bladder. The fibrin/fibrinogen degradation products in human urine samples have been used as a bladder cancer marker [59]. The leakage of fibrin is regulated by the blood–urine barrier in bladder epithelial cells. This suggests that damage to the blood urine barrier is associated with bladder cancer initiation and that PRELP may have the ability to regulate the blood–urine barrier. Interestingly, we have found that in *OMD*^−/−^ and *PRELP*^−/−^ mice, umbrella cells are connected to each other by adherens junctions. It is known that the loss of E-cadherin is a marker of conversion from benign to malignant bladder cancer. Thus, double knockout of *OMD/PRELP* and E-cadherin may reveal the process of malignant cancer initiation.

### 3.3. EMT/MET Regulated by OMD and PRELP

During malignant transformation, cancer cells have acquired mesenchymal-like characteristics such as anoikis resistance and invade adjacent tissues. Our results showed that OMD or PRELP overexpression in bladder cancer cells resulted in an increase of epithelial-like properties such as tight junction induction and adherens junction activation as well as a change of EMT markers. A cardinal feature of cancer is the ability for anchorage-independent growth, which changes the properties of cell–cell and cell–matrix adhesion conferred at EMT.

Umbrella cells secrete signaling proteins such as EGF and TGF-β [60]. The concept that OMD or PRELP mediated the inhibitory activity of TGF-β and EGF pathways could be important for the regulation of EMT/MET, because the TGF-β/Smad2 pathway is the biggest common target of all SLRP family members [2] and is a well-known regulator of EMT/MET [46]. In addition, EGF is known as a major regulator of EMT/MET [46] and is one of the most established targets of cancer treatment [61]. Previously, we reported that the simultaneous regulation of Xnr2, FGF, and BMP pathways by Tsukushi, another SLRP member, had an increased synergistic effect compared to the single regulation of each pathway alone [6].

OMD and PRELP are selectively expressed in the ciliary body of the retina and in ependymal cells in the brain (paper in preparation) that are characterized by strong tight junctions forming the blood–CSF barrier. The expression of many components of tight junctions is associated with tumorigenesis [62]. However, so far, there is no report showing that the knockout of any tight junction component by itself can spontaneously lead to tumor formation, although, hyperplasia of the gastric epithelium has been observed in an occludin knockout model [63]. This suggests that the loss of tight junctions alone is not sufficient to initiate bladder cancer. TGF-β and EGF pathways are involved in the regulation of many cancer-associated signaling pathways, suggesting that in addition to the loss of tight junctions in an *OMD*^−/−^ or *PRELP*^−/−^ bladder, further regulation of TGF-β and EGF downstream signaling components might be required for cancer initiation. Of note, one limitation of our study is that although the TGF-β-flag protein bound with OMDmyc and PRELPmyc proteins directly, the binding affinities of secreted TGF-β to the OMD and PRELP is unknown; therefore, further studies are required.

### 3.4. The Similarity and Difference between OMD and PRELP

Both OMD and PRELP were downregulated, especially in bladder cancer. Our results indicated that although OMD and PRELP share considerable amount of signal pathways, there are some differences in the observed phenotypes: branching, proliferation, bladder cancer progression, and protein expression. Functional difference between OMD and PRELP may be associated with certain cancer phenotypes. This indicates that they would play a redundant and non-redundant function in bladder cancer.

### 3.5. Diagnostic and Therapeutic Potential of OMD and PRELP in Bladder Cancer

*OMD* and *PRELP* are expressed in normal human epithelia. However, in many epithelial cancers, they are strongly downregulated. Particularly, their expression in the bladder is drastically reduced even in very early stages of cancer. This potentially means that it is possible to classify a patient’s clinical state based solely on their OMD and PRELP expression status from early-stage cancers. So far, several diagnostic markers of bladder cancer have been used in clinics such as BTA-Stat (sensitivity 50–70%, specificity 67–78%) and fibrin degradation products (FDP) (sensitivity 52–68.4%, specificity 79.6–91%) [64]. With our findings, we show that the assessment of *OMD* and *PRELP* expression status can be used as a novel, more sensitive, criterion in assessing the initiation and progression of bladder cancer. We also observed a similar evaluation in renal cell carcinoma and retinoblastoma (paper in preparation), proposing their diagnostic potential in various epithelial cancers, possibly through using new technology such as quench bodies to detect loss-of-function regions [65]. This study demonstrates that the functions of OMD and PRELP are partially redundant in the regulation of both cell–cell integrity and cancer initiation/progression, and they are potentially important, especially for bladder cell therapeutics.

## 4. Materials and Methods

Materials and Methods are described in the Supplementary Materials and Methods. The accession number for the raw and processed data of microarray and RNA-seq data from *OMD* and *PRELP* knockdown experiments reported in this paper is GEO: GSE63955 and GSE144295. Other data supporting our findings can be found either in this article or in the supplementary materials. Please contact the corresponding author for all “unpublished data” and “paper in preparation” requests.

The research protocol was reviewed and approved by the Ethical Committee of Addenbrooke’s Hospital, Cambridgeshire Local Research Ethics Committee (No. 03/018).

## 5. Conclusions

In this study, we demonstrated that two SLRP proteins, OMD and PRELP, are novel activators of the cell–cell integrity by inhibiting EMT through the simultaneous inhibition of TGF-β and EGF signaling. The downregulation of OMD and PRELP expression was observed in all of the cancers we analyzed, including bladder cancer. We showed that in association with a change of EMT states, OMD or PRELP suppression in mice resulted in an initiation of bladder cancer, while the activation of OMD or PRELP inhibited bladder cancer progression in vitro and in vivo. We propose that OMD and PRELP-mediated regulation of EMT is important for the initiation of human bladder cancer.

## Supplementary Materials

The following are available online at https://www.mdpi.com/2072-6694/12/11/3362/s1, Figure S1: Microarray analysis of *OMD* and *PRELP* expression in various cancers and normal tissues; Figure S2: Expression analysis of OMD and PRELP in various human cancers using Gene Logic Inc. Figure S3: *OMD* and *PRELP* expression analysis in various cancer cells and ontological analysis of expression profiling data; Figure S4: Effect of OMD or PRELP on cell properties under standard cell culture conditions; Figure S5: Quantification of OMD and PRELP effects; Figure S6: PRELP is expressed in subpopulation of bladder umbrella epithelial cells; Figure S7: The ontological analysis in *OMD*^−/−^ and *PRELP*^−/−^ bladder epithelia; Figure S8: Original Western Blotting images used in Figure 5.; Table S1: Statistical analysis of *OMD* and *PRELP* expression levels in clinical bladder tissues; Table S2: Relationship between *OMD* and *PRELP* expression levels and carcinogenesis; Table S3: Primer sequences for quantitative RT-PCR.
