# Supplementary material for: Two Secreted Proteoglycans, Activators of Urothelial Cell–Cell Adhesion, Negatively Contribute to Bladder Cancer Initiation and Progression
Source: Cancers (Basel). 2020 Nov 13;12(11):3362. doi: 10.3390/cancers12113362 (PMC7697838; doi:10.3390/cancers12113362)
Supplement: Supplementary file 1 [file cancers-12-03362-s001.pdf]

Supplementary Materials: Two Secreted Proteoglycans, Activators of Urothelial Cell–Cell Adhesion, Negatively Contribute to Bladder Cancer Initiation and Progression

Vasiliki Papadaki, Ken Asada, Julie K. Watson, Toshiya Tamura, Alex Leung, Jack Hopkins, Margaret Dellett, Noriaki Sasai, Hongorzul Davaapil, Serena Nik-Zainal, Rebecca Longbottom, Makoto Nakakido, Ryo Torii, Abhi Veerakumarasivam, Syuzo Kaneko, Mandeep S. Sagoo, Gillian Murphy, Akihisa Mitani, Kohei Tsumoto, John D. Kelly, Ryuji Hamamoto and Shin-ichi Ohnuma

1. Supplementary Materials and Methods

1.1. Antibodies

Anti-Myc tag (05–724 and 9E10) were purchased from Upstate. Anti-p38 mitogen-activated protein kinase (MAPK) (#9212), Phospho-p38 MAPK Thr180/Tyr182 (#9211), anti-β-catenin (#8480), anti-epidermal growth factor receptor (EGFR) (#4267), anti-phospho-insulin like growth factor (IGF)-1 Receptor (#3918), anti-IGF-1 Receptor (#9750), anti-phospho-Akt (#4060), anti-Akt (#4691), anti-phospho-extracellular signal-regulated kinase (ERK)1/2 (#4370), anti-ERK1/2 (#4695), anti-Smad1 (#6944), anti-phospho-Smad1 (#9511), anti-Smad2 (#5339), anti-phospho-Smad2 (#3101), anti-glycogen synthase kinase (GSK)-3β (#9315), anti-phospho-GSK-3β (#5558), anti-protein kinase-Cα(PKCα) (#2056), anti-hepatocyte nuclear factor 4α (HNF4α) (#3113), anti-Myc (#2276, Upstate), and anti-β-actin (#4970) antibodies were from Cell Signaling Technology except.

1.2. Sample Collection of OMD and PRELP Expression in British Samples

Specimens of urothelial cell carcinoma and renal cell carcinoma were collected during surgery and snap frozen in liquid nitrogen. Specimens of normal bladder urothelium and kidney tissues were collected from patients with no evidence of malignancy. Use of tissues for this study was approved by Cambridgeshire Local Research Ethics Committee (#03/018).

1.3. Quantitative Analysis of OMD and PRELP Expression in British Samples

We used 123 bladder cancer tissues and 23 normal bladder tissues in Cambridge Addenbrooke's Hospital. Five 7 μm sections were cut from each tissue for laser capture microdissection and two 7 μm "sandwich" sections were sectioned, stained, and assessed for cellularity and tumor grade by an independent consultant urohistopathologist. RNA was extracted using an RNeasy Micro Kit (Qiagen, Crawley, UK) or TRI Reagent™ (Sigma, Dorset, UK. qRT-PCR analysis was performed using the ABI prism 7700 Sequence Detection System (Applied Biosystems, Warrington, UK). The primers used are tabled in Supplementary information, Table S3.

1.4. OMD and PRELP Expression Analysis in MIBC Cell Lines and NMIBC Patient Samples

Gene expression data of MIBC cell lines are available in NCBI GEO (GSE97768). The expression data were processed by authors using the scaling factor normalization method. We performed log transformation to all data before conducting the expression analysis. The expression levels of *APP, CHEK1, EGFR, ERBB2, TP53, TUBA1C, TUBB1,* and *TUBD1* were compared with the expression levels of *OMD* and *PRELP*. The expression data of NMIBC

patient samples, E-MTAB-4321, were downloaded from ArrayExpress (https://www.ebi.ac.uk/arrayexpress/). We performed log transformation to all fragments per kilobase of transcription per million mapped reads (FPKM) values data before conducting the expression analysis. The expression levels of *APP*, *CHEK1*, *EGFR*, *ERBB2*, *TP53*, *TUBA1C*, and *TUBD1* were compared with the expression levels of *OMD* and *PRELP*.

1.5. OMD Mutation Analysis Using the Cancer Genome Atlas (TCGA) Dataset

A total of 3,142,246 somatic substitutions/indels were interrogated from 33,096 primary human cancers (33 different tissue-types). The data were downloaded from the TCGA (https://tcga-data.nci.nih.gov/tcga/) on January 28th, 2019. The number of 3,142,246 is the unique mutations added up in the TCGA genomic data commons (GDC).

1.6. Xenograft

EJ28 bladder cancer cell lines (EJ28/wt) and OMD stably expressed EJ28 cell lines (EJ28/OMD) xenografts were initiated by the subcutaneous implantation of 5×10^6 cells, suspended in 100 μl of MATRIGEL (Becton Dickinson, USA) solution, into the right flank of nude mice in accordance with UK Home Office regulations and allowed to develop to measurable size. Two weeks after inoculation, tumor measurement was started. Tumor volume was assessed using digital calipers. The volume was calculated using the following formula.

$$\text{Tumor volume (mm}^3\text{)} = (a)\times (b)^2 / 2 \quad (1)$$

(a); longer diameter, (b); shorter diameter.

1.7. Mathematical Model of Umbrella Layer Breakdown

The epithelial layers were modeled first by constructing an idealized model of epithelial cells (width 20 ◎m× height 10 ◎m) on a straight substrate. The substrate was deformed such that the substrate is deformed by one cell height over 3 cell widths, both in convex and concave. The elasticity of the cells was assumed to be uniform and set to 1.6 kPa (Guz N et al., 2014), and all cell junctions were tightly connected. The model was generated using the commercial computational modelling suite ANSYS Workbench version 19.2 (ANSYS Inc., Cannonsburg, USA). Then, this model deformation was computed using finite element analysis, and the results was visualized in terms of the force (contact pressure) at the junctions.

1.8. Transwell Assay

The assay insert plates (BD Bioscience) were prepared by rehydrating the Matrigel™ coating with phosphate-buffered saline for 2 h at 37 °C. The rehydration solution was carefully removed, 0.75 mL Dulbecco's modified Eagle medium (DMEM) containing 10% fetal bovine serum (FBS) was added to the plate well as a chemoattractant and 0.5 mL of cell suspension (2.5×10^4 cells) in 0.1% FBS contained DMEM was added to each insert well. The assay insert plates were incubated for 48 h at 37 °C. Following incubation, the medium was removed from the upper chamber, and the insert membrane was stained with hematoxylin using Hemacolor® (MERCK, Watford United Kingdom). After staining, cells attached to the top of membrane were wiped off, and the dye staining cells under the membrane was eluted with 0.1 M citric acid (unbuffered) and read absorbance at 630 nm.

1.9. Immunostaining of Cultured Cells

For the antibodies against occludin, cingulin, and ZO-1, cells were fixed in methanol for 10 min at −20 °C. For the antibodies against E-cadherin, vimentin, and β-catenin, cells were fixed in 4% paraformaldehyde (PFA) for 15 min at room temperature followed by a 5 min incubation in 0.5% Triton X-100 in blocking buffer (phosphate-buffered saline (PBS) containing

10% goat serum). The samples were washed and incubated for 1 h in blocking buffer. After blocking and incubation with primary antibodies for 16 h at 4 °C, the samples were incubated with the secondary AlexaFluor 488 (1:500, Life Technologies, ThermoFisher, Dartford United Kingdom) in blocking buffer for 1 h at room temperature. The following antibodies were used: occludin (1:100, #33–1500, Life technologies), ZO-1 (1:100), cingulin (1:400, #374300, Invitrogen) and β-catenin (#8480), E-cadherin (#3195), and vimentin (1:100, #5741) are from Cell Signaling Technology.

1.10. Matrigel Invasion Assay

EJ28 bladder cancer cells and OMD or PRELP stably expressed EJ28 cells were plated on top of a layer of growth factor-reduced Matrigel (BD Bioscience,). Eight-well multi-chamber coverslips were covered with 100 μL Matrigel and left to gel for 45 min at 37 °C. Then, 10,000 cells in 200 μL of Matrigel were plated and cultured with DMEM containing 10% FBS. The medium was replaced with fresh medium twice a week. After 7 days, 3D morphogenesis was assessed by microscopic images.

1.11. Electron Microscopy

Cells or segments of bladders were fixed in 3% glutaraldehyde/1% paraformaldehyde in 0.08 M sodium cacodylate-HCl buffer (pH 7.4) and after washing with PBS were immersed in 1% aqueous osmium tetroxide solution for 2 h at room temperature. Samples were washed again and dehydrated by single 15 min. incubations in 50%, 70%, 90%, and 100% ethanol (3×), 2× 20 min changes of propylene oxide and left overnight in a 1:1 mixture of propylene oxide:araldite for the solution to infiltrate. Afterwards, samples were transferred to araldite resin, rotating for 6 h to remove any traces of propylene oxide prior to embedding. Finally, they were embedded in fresh resin for 24 h at 60 °C. Semithin sections were cut using a Leica ultracut S microtome with a diamond knife, were stained with a mixture of 1% borax and 1% toluidine blue in 50% ethanol at 60 °C, and after drying were mounted in dibutylphthalate polystyrene xylene (DPX). Ultrathin sections were cut at 60 or 100 nm, stained with lead citrate and viewed in a JEOL 101 TEM operating at 100 kV.

1.12. Immunohistochemistry and Bladder Analysis

Mouse bladders were isolated and fixed in 4% PFA for at least 24 h before paraffin embedding and sectioning. Then, 5 μm sections were collected on poly L-lysine slides, and 5 slides per sample were stained for hematoxylin and eosin (H&E). The quantification of epithelial bursts was performed on 5 samples per bladder. For immunostaining, slides were dewaxed for 10 min in Histoclear and rehydrated in an ethanol–water graded series. Antigen retrieval was performed by boiling the samples for 15 min in citrate (pH 6.0) or Tris-ethylenediaminetetraacetic acid (EDTA) buffer (pH 9.0) depending on the antibody. Sections were blocked for 1 h in 10% goat serum in PBS and were incubated overnight with primary antibodies at 4 °C. Detection was performed by incubation with fluorescent anti-rabbit or anti-mouse AlexaFluor 488 secondary antibodies for 1 h at room temperature (1:500 dilution, Life technologies). The primary antibodies used were anti-fibrinogen (1:1000, Abcam), anti-Ki67 (1:100, Abcam), anti-ZO-1 (1:100, produced by Karl Matter, IOO, UCL), uroplakin III (1:100, Fitzgerald), and E-cadherin (1:400, Cell Signaling Technology). For fibrin detection in the mouse bladders, tinctorial Martius, Scarlet and Blue (MSB) staining was performed under the standard method.

1.13. Mouse Urine Testing

Mouse urine was tested for blood and protein using Fisherbrand™ Urine Reagent Strips (Thermo Fisher Scientific). Drops of urine were loaded onto the appropriate pads on the strips, and measurements were calculated within 1 min, depending on the coloration of each pad.

1.14. Quantification and Statistical Analysis

Error bars in all graphs in the figures indicate error of means. Student-t test values are * < p = 0.01, ** < p = 0.005, and *** < p = 0.001. Always, n is more than three. For grade correlation studies, two-tailed Spearman's rank correlation was performed. To determine the significance of differential expression in the laser captured tissue, a two-sided Mann–Whitney *U* nonparametric analysis was performed. Statistical evaluations were done using the STATA (version 8.0; StataCorp, College Station, TX, USA) and StatView (version 5.0; SAS, Cary, NC, USA). The cutoff values to distinguish tumor from normal were determined through calculation of the interquartile range (IQR). Subtracting the first quartile $\times_{.25}$) from the third quartile ($\times_{.75}$) in each data was calculated to derive a cutoff value. Any data observation that lies more than 1.5*IQR lower than the first quartile or 1.5*IQR higher than the third quartile was an outlier and derived a cutoff value as follows: Cutoff = [(smallest non-outlier observation in normal tissues) + (largest non-outlier observation in tumor tissues)]/2.

1.15. In Silico Expression Analysis

BioExpressTM database (Gene Logic, Gaithersburg, MD) and Oncomine (Life technologies, http://www.oncomine.org) were analyzed, and the box plots were generated for OMD and PRELP with the normalized gene expression data extracted. RNA was prepared and gene expression analysis was determined at Gene Logic Inc. using Affymetrix GeneChip® HG-U133Plus2 microarrays containing oligodeoxynucleotides that correspond to approximately 40,000 genes/ESTs. Oncomine data were log transformed, median centered per array, and the standard deviation was normalized to one per array. The Oncomine Cancer Microarray database was used to study gene expressions of OMD and PRELP in 40 human tumor types and their normal tissue counterpart as described previously (Kassambara et al., 2009; Rhodes et al., 2007; Rhodes et al., 2004). All data were log transformed, median centered per array, and the standard deviation was normalized to one per array (Rhodes et al., 2007; Rhodes et al., 2004). *t*-Tests were conducted both as two-sided for differential expression analysis and one-sided for specific overexpression analysis. For the purpose of the whole study, p values were corrected for multiple comparisons by the method of false discovery rates (Rhodes et al., 2004).

1.16. Construction of Stable Cell Lines and Cell Biological Analyses

Expression constructs of OMD, OMD-myc, PRELP, and PRELP-myc were constructed using pIRES2EGFP vector. EJ28 cells were transfected with OMD, OMD-myc, PRELP, or PRELP-myc using Lipofectamine 2000 (Invitrogen). After 48 h, transfected cells were selected by treating with 750 μg/mL G418 (Invitrogen) for two weeks. Then, 1% of the survived cells were GFP+, and flow cytometry was performed to enrich GFP+ cells (FACS Aria SORP instrument, BD). Then, individual GFP+ cells were isolated by a serial dilution, and clonal populations were obtained. Multiple clones were derived for each plasmid transfected to control for any effects that might be due to the integration site. The primers used for the analysis of OMD and PRELP expression were tabled in the Supplementary Information, Table S3.

1.17. OMD$^{-/-}$ and PRELP$^{-/-}$ Mice

OMD and PRELP knockout mice were generated by the Takeda Pharmaceutical Company, and wild-type and heterozygote founders were imported to our animal facility. Mice were kept in individually ventilated cages (IVCs) in a 12 h light:dark cycle, were fed a

complete pelleted mouse diet, and had constant access to water. *OMD* or *PRELP* flox ES cells were generated from C57BL/6J ES cells by homologous recombination with the targeting vectors, which were constructed by insertion of the first LoxP sequence and insertion of the neomycin resistant unit. Cre expression plasmid was electroporated into the recombinant flox ES cells to generate ES cells harboring knockout alleles. The resulting knockout ES cells were injected into ICR tetraploid blastocysts to generate chimeric male mice, and the chimeric mice were backcrossed to C57BL/6J females. Single knockout mice (*OMDLacZ/LacZ* and *PRELPLacZ/LacZ*) and their double knockout (*OMDLacZ/LacZPRELPLacZ/LacZ*) were generated by cross breeding within the colony. Genotyping the PCR of genetically modified and control mice was performed using ear punches of the animals. All animal procedures were performed in accordance to the Animals (Scientific procedures) Act 1986 of the UK Government. All mice were housed in compliance with the Home Office Code of Practice.

1.18. Cell Morphology

The stable EJ28 subcell lines were plated out onto coverslips placed in 6-well dishes and incubated for 48 h to allow cellular adherence. For phalloidin, the cells were fixed in 4% paraformaldehyde (PFA) for 10 min, permeabilized in 0.1% Triton X-100. A 1:40 dilution of Alexafluor-555-conjugated phalloidin (Thermo Fisher Scientific. Dartford, United Kingdom) was applied, and samples were incubated for 2 h in the dark. For tubulin staining, cells were fixed in methanol at −20 °C and incubated with tubulin antibody (DM1α, Sigma-Aldrich, 1:1000) and subsequent alexafluor-555-conjugated anti-mouse antibody (Thermo Fisher Scientific, London, United Kingdom.

1.19. Cell Cycle Analysis

Samples were trypsinized and resupended in 500 µL cold PBS. Then, the suspension was mixed with 4.5 mL 70% ethanol and left at 4 °C for 2 h to fix the cells. Then, cells were pelleted and resuspended in staining buffer (25 mM Tris, 0.5 M NaCl, 100 µg/mL RNase A, 50 µg/mL propidium iodide (PI)), and incubated for 15 min at 37 °C in the dark. Then, cells were passed through filter-top flow cytometry tubes (BD Falcon) and examined on a FACS Calibur instrument (Becton Dickinson, Swindon United Kingdom). An unstained control was included in the analysis for technical optimization. Data were analyzed using FlowJo software.

1.20. Growths Curves Analysis

At each time point, samples were trypsinized, and the resultant suspended cells were counted on a Vicell automated cell counter (Beckman Coulter, High Wycombe United Kingdom). A non-linear regression analysis was applied to the data to establish doubling times, goodness of fit, and 95% confidence intervals.

1.21. Proliferation Analysis

Cells were seeded the day before experiments, and cells were refilled with growth medium containing 10 µM BrdU next day. After 2 h, the cells were trypsinized, washed, and resuspended in 500 µL PBS. The resuspended cells were added to 4.5 mL 70% ethanol and fixed at 4 °C in the dark. Fixed cells were spun down, resuspended in 100 µL distilled water, and incubated at 85 °C for 5 min. After 2 min cooling on ice, the cells were spun down and resuspended in 100 µL antibody binding buffer (PBS + 1% BSA (w/v) + 0.1% Triton X-100 + 5% alexafluor-647 conjugated anti-BrdU antibody (v/v) (Thermo Fisher Scientific)) for 30 min at room temperature in the dark. Cells were spun down and resuspended in 1 mL PBS + 100 µg/mL RNase A + 10 µg/mL propidium iodide; then, they were incubated for 30 min at room temperature in the dark. Then, cells were passed through filter-top flow cytometry tubes and

examined on a FACS Calibur instrument. A total of 30,000 events were recorded for each sample. Data were analyzed using FlowJo software.

1.22. Annexin Assay

Cells were seeded the day before experiments and were refilled with growth medium or growth medium supplemented with 1 μg/mL Mitomycin C. Then, 24 h after treatment, samples were resuspended in 100 μL annexin binding buffer (10 mM 4-(2-hydroxyethyl)-1-piperazineethanesulfonic acid (HEPES), 140 mM NaCl, 2.4 mM $CaCl_2$, pH 7.4) with annexfluor-647 conjugated annexin and 1 μg/mL propidium iodide. Samples were incubated for 15 min in the dark; then, they were diluted with 400 μL annexin binding buffer, passed through filter-top flow cytometry tubes, and examined on a FACS Calibur instrument. A total of 10,000 events were recorded for each sample. Data were analyzed using FlowJo software (https://www.flowjo.com).

1.23. Soft Agar Assays

Twice the usual amount of serum and antibiotics was added to this solution and mixed with the 1.2% agarose solution to yield a final 1× growth medium + 0.6% agarose solution. A sterile solution of 0.6% agarose was mixed with the aforementioned 2× growth medium to yield 1× growth medium + 0.3% agarose. A suspension of 3000 cells in 200 μL 1× growth medium was added to a 1 mL aliquot of the 1× growth medium + 0.3% agarose solution, and the resultant mixture was plated out in a 6-well dish, on top of the 1× growth medium + 0.6% agarose layer that was prepared this way. The plates were incubated for two weeks until visible colonies appeared. Colonies were imaged with a phase-contrast microscope and counted.

1.24. Scratch Assay

Twelve-well dishes were inverted, and three horizontal lines were drawn across each well with a marker pen. Stable cell lines were plated in 12-well dishes and grown to confluence. Using a ruler and a 10 μL pipette tip, a vertical and linear scratch wound was created in each well. Using the lines drawn with a marker pen for orientation, a brightfield photograph was taken through a microscope of three areas of each scratch at each time point. The surface area of the scratch wound in each image was measured using ImageJ software, and the percentage invasion at each time point was calculated.

1.25. Cdc42 Activation Assay

Cells were seeded at 1×10^5 cells/well in the 10% FBS containing DMEM in a 24-well plate and after 24 h, cells were harvested and analyzed for levels of active Cdc42 using the G-LISA assay kits (Cytoskeleton).

1.26. Microarray Hybridization and Statistical Analysis for the Clarification of the Pathways Influenced by OMD and PRELP

The T-Rex-293T system (Life technologies) was used to make cell lines expressing OMD or PRELP. The 5637 cell line was transfected with siOMD, siPRELP, siEGFP, or siFFLuc. Purified total RNA was labeled and hybridized onto Affymetrix U133 Plus 2.0 GeneChip oligonucleotide arrays (Affymetrix, Santa Clara, CA), and the detailed method was described previously (Hayami et al., 2011; Hayami et al., 2010). Pathway analysis of differential expression data was carried out using Ingenuity Pathway Analysis software (Ingenuity Systems). Detail: To identify the genes pathways influenced by OMD and/or PRELP, transcriptome analysis was performed at eight conditions; OMD overexpression in T-REx-293T cells, PRELP overexpression in T-REx-293T cells, two controls T-REx-293T cells (Mock and CAT), OMD knockdown in the 5637 cells, PRELP knockdown in the 5637 cells, and two controls

(siEGFP and siFFLuc). Each experiment was performed at least three times. Probe signal intensities were normalized by RMA and quantile normalization methods using R and Bioconductor. Signal intensity fluctuation due to inter-experimental variation was estimated. Each experiment was replicated (1 and 2), and the standard deviation (stdev) of $\log_2(\text{intensity}_2/\text{intensity}_1)$ was calculated for each of a set of intensity ranges with the midpoints being at $\log_2((\text{intensity}_1 + \text{intensity}_2) / 2) = 5, 7, 9, 11, 13$, and 15. We modeled the intensity variation using the formula $\text{stdev}(\log_2(\text{intensity}_2/\text{intensity}_1)) = a * (\log_2((\text{intensity}_1 + \text{intensity}_2) / 2)) + b$ and estimated parameters a and b using the method of least squares. The standard deviation of intensity fluctuation was calculated using these values. The signal intensities of each probe were compared between experimental cases (siOMD/siPRELP [knockdown] or T-REx-293T-OMD/PRELP [overexpression]) and control cases (siEGFP/siFFLuc [knockdown] or T-REx-293T-Mock/CAT [overexpression]) and tested for up/down-regulation by calculating the z-score: $\log_2(\text{intensity}_{\text{EXP}}/\text{intensity}_{\text{CONT}}) / (a * (\log_2((\text{intensity}_{\text{EXP}} + \text{intensity}_{\text{CONT}}) / 2)) + b)$. Resultant p values for the replication sets were multiplied to calculate the final p value of each probe. These procedures were applied to each comparison: siEGFP vs. siOMD or siPRELP, siFFLuc vs. siEGFP or siOMD, T-REx-293T-Mock vs. T-REx-293T-OMD or T-REx-293T-PRELP, T-REx-293T-CAT vs. T-REx-293T-OMD or T-REx-293T-PRELP, siEGFP vs. siFFLuc, and T-REx-293T-Mock vs. T-REx-293T-CAT, respectively. Up/downregulated gene sets were those that simultaneously satisfied the following criteria: (1) The Benjamini–Hochberg false discovery rate (FDR) ≤ 0.05 for siEGFP vs. siOMD or siPRELP, T-REx-293T-Mock vs. T-REx-293T-OMD or T-REx-293T-PRELP, (2) FDR ≤ 0.05 for siFFLuc vs. siOMD or siPRELP, T-REx-293T-CAT vs. T-REx-293T-OMD or T-REx-293T-PRELP, and the regulation direction is the same as (1), and (3) siEGFP vs. siFFLuc or T-REx-293T-Mock vs. T-REx-293T-CAT has the direction opposite to (1) and (2) or $p > 0.05$ for siEGFP vs. siFFLuc or T-REx-293T-Mock vs. T-REx-293T-CAT. A pathway analysis using the hyper-geometric distribution test, which calculates the probability of overlap between the up/downregulated gene set and each GO category compared against another gene list that was randomly sampled. The identified up/downregulated genes were tested to determine whether they are significantly enriched (FDR ≤ 0.05) in each category of "Biological processes" (857 categories) as defined by the Gene Ontology database. The siRNA used were summarized in Supplementary Information, Table S3. The control siEGF and siFFLuc have been previously described (Hayami et al., 2010 and Hayami et al., 2011).

1.27. Mouse Tissue Cryosectioning

Tissues from mice were fixed in 4% PFA for different amounts of time depending on the size. After fixation, tissues were washed in PBS, transferred to 30% sucrose (Sigma, UK) solution, and kept there until they sunk. They were embedded in OCT compound (Tissue Tek, Sakura Finetek) and were snap frozen in a dryice/isopentane bath. Frozen tissues were stored at −80 °C until use. Then, 10–18 microns serial sections were cut with Lica CM3050 cryostat, mounted onto Superfrost Plus glass slide (Fisher Scientific, UK), and stored at −80 °C until use.

1.28. Mouse Paraffin Embedding of Tissues and Sectioning

Tissues for paraffin sectioning were processed in the Institute's Pathology department using an automated machine (Lica ASP300S). Wax sections were transferred in a 40 °C water bath and mounted onto superfrost slides. The excess water was dried off the slides, and they were put on a hot plate (around 60 °C) for 30 min. Slide were stored at room temperature.

1.29. β-Galactosidase Analysis

Mouse bladders were isolated from 3-month adult mice and were fixed with 4% PFA at 4 °C briefly for 1 h. Afterwards, they were washed in PBS and left at 30% sucrose until they had sank, and they were subsequently frozen in OCT. Cryosections at 10 μm were collected, post-

fixed for 10 min in 4% PFA, washed two times for 20 min with PBS, and stained overnight in the X-gal solution at 37 °C. For expression analysis, immunofluorescence was performed on top of the X-gal staining. Samples were washed from the X-gal solution, immediately blocked for 1 h with 10% goat serum, and incubated with the primary antibodies overnight. Secondary staining was done using goat anti-rabbit or goat-anti mouse AlexFluo-488 antibodies. The following primary antibodies were used: Laminin (1:200, Abcam), CK14 (1:100, Covance), CD44 (1:50, Proteintech), p63 (1:100, Santa Cruz), uroplakin III (1:100, Fitzgerald), CK18 (1:100 Abcam), CK5 (1:100, Abcam).

1.30. Hematoxylin and Eosin Staining and Special Stains

H&E staining was performed in an automated system in the Pathology department. Paraffin sections were de-waxed in xylene and passed through two changes of absolute alcohol, two changes of 90% alcohol, and one change of distilled water; then, they were stained in Harris hematoxylin for five changes. Samples were washed in running tap water for one change, differentiated in 1% acid alcohol for one change, washed again in tap water for one change, passed through two changes of 90% alcohol, counterstained with eosin for three changes, dehydrated in 95% alcohol for two changes, and cleared with xylene. Samples were mounted using DPX. Special stains, including von Kossa, alcian blue, congo red, and MSB, were also performed in the Pathology department following the department's specific protocol. Methylene blue and basic fuchsin staining was performed on semi-thin sections of xenografted tumors. Then, slides were covered with basic fuchsin solution for 2 min at room temperature. Finally, they were rinsed with dH_2O and mounted with DPX.

1.31. Expression Profiling of Bladder Epithelial Layer

Four wild-type mice, three *OMD*$^{-/-}$, and three *PRELP*$^{-/-}$ knockout mice bladder samples were used for RNA-seq analysis. RNA was extracted via a QIAGEN RNAeasy Mini kit. Bladder tissue samples were excised and homogenized using a rotor–stator homogenizer. RNA samples were processed using the KAPA mRNA HyperPrep Kit (Roche KK8580) and mRNA was isolated from total RNA using Oligo dT beads to pull down poly-adenylated transcripts. The purified mRNA was fragmented using chemical hydrolysis (heat and divalent metal cation) and primed with random hexamers. Strand-specific first-strand cDNA was generated using Reverse Transcriptase in the presence of Actinomycin D to prevent spurious DNA-dependent synthesis. The second cDNA strand was synthesized using dUTP in place of dTTP to mark the second strand. Then, the resultant cDNA is "A-tailed" at the 3′ end to prevent self-ligation and adapter dimerization. Full-length xGen adaptors (IDT), containing two unique 8bp sample specific indexes, a unique molecular identifier (N8), and a T overhang are ligated to the A-Tailed cDNA. Then, successfully ligated cDNA molecules were enriched with a limited cycle PCR (50 ng of starting material, 15 PCR cycles). Samples were sequenced on the NextSeq 500 instrument (Illumina, San Diego, US). Data were demultiplexed and converted to fastq files using Illumina's bcl2fastq Conversion Software v2.19. Fastq files were aligned to the Mus musculus genome GRCm38 using RNA-STAR 2.5.2b and then UMI deduplicated using Je-suite (1.2.1). Reads per transcript were counted using FeatureCounts, and differential expression was estimated using the BioConductor package SARTools, which is a DESeq2 wrapper. Log2 fold change and *p* values of pairwise differential expression between wild-type samples and knockout samples were analyzed using Qiagen's Ingenuity Pathway Analysis (version 48207413), creating diagrams and datasets for related canonical pathways, disease mechanisms, and affected molecules.

Table 1. Statistical analysis of OMD and PRELP expression levels in clinical bladder tissues.

	OMD				PRELP			
**Characteristic**	***n***	**Mean**	**SD**	**95%CI**	***n***	**Mean**	**SD**	**95%CI**
Normal (Control)	31	4398	3.605	3.076–5.721	31	1.674	0.939	1.324–2.025
Tumor (Total)	126	0.420	1.290	0.193–0.648	126	0.215	0.557	0.127–0.407
Tumor stage								
pTa, pT1	90	0.452	1.466	0.145–0.759	90	0.259	0.647	0.124–0.395
pT2	26	0.433	0.772	0.121–0.745	26	0.121	0.183	0.047–0.195
pT3, pT4	7	0.008	0.022	−0.012–0.028	7	0.024	0.033	−0.006–0.054
Tumor grade								
G1	12	1.127	3.267	−0.948–3.203	12	0.498	0.873	−0.057–1.053
G2	63	0.280	0.763	0.088–0.472	63	0.210	0.635	0.050–0.370
G3	50	0.436	0.981	0.157–0.715	50	0.157	0.297	0.072–0.241
Metastasis								
Negative	99	0.484	1.430	0.197–0.771	99	0.252	0.619	0.128–0.375
Positive	27	0.185	0.352	0.062–0.334	27	0.080	0.163	0.015–0.144
Gender								
Male	91	0.496	1.467	0.191–0.802	91	0.225	0.548	0.110–0.339
Female	32	0.244	0.633	0.015–0.472	32	0.179	0.614	−0.042–0.401
Recurrence								
No	28	0.167	0.447	−0.007–0.340	28	0.137	0.350	0.001–0.273
Yes	51	0.384	1.122	0.069–0.700	51	0.145	0.375	0.039–0.250
Died	8	0.141	0.271	−0.086–0.367	8	0.075	0.085	0.003–0.146

Table S2: Relationship between *OMD* and *PRELP* expression levels and carcinogenesis.

Characteristic	Normal		Tumor (Total)		Tumor (Early stage[a])		Tumor (Advanced and Late Stages[b])	
	n	Specificity (%)	*n*	Sensitivity (%)	*n*	Sensitivity (%)	*n*	Sensitivity (%)
**Bladder**								
OMD (cutoff: 0.897)								
Above the cutoff	26	83.9	14	88.9	10	88.9	4	88.9
Below the cutoff	5		112		80		32	
PRELP (cutoff: 0.415)								
Above the cutoff	28	90.3	12	90.5	10	88.9	2	94.4
Below the cutoff	3		114		80		34	
Combined OMD and PRELP								
Both above the cutoff	26	83.9	6	95.2	5	94.4	1	97.2
At least one below the cutoff	5		120		85		35	
At least one above the cutoff	31	100	20	84.1	15	83.3	5	86.1
Both below the cutoff	0		106		75		75	

[a] Early stage: pTa and pT1, [b]. pT2, pT3 and pT4.

Table S3: Primer sequences for quantitative RT-PCR.

Gene Name	Primer Sequence
GAPDH (housekeeping gene)-f	5′ GCAAATTCCATGGCACCGTC 3′
GAPDH (housekeeping gene)-r	5′ TCGCCCCACTTGATTTTGG 3′
SDH (housekeeping gene)-f	5′ TGGGAACAAGAGGGCATCTG 3′
SDH (housekeeping gene)-r	5′ CCACCACTGCATCAAATTCATG 3′
OMD-f	5′ GCAAATTCCATGGCACCGTC 3′
OMD-r	5′ TCGCCCCACTTGATTTTGG 3′
PRELP-f	5′ CTGTCCCACAACAGGATCAGCAG 3′
PRELP-r	5′ CAGGTCCGAGGAGAAGTCATGG 3′

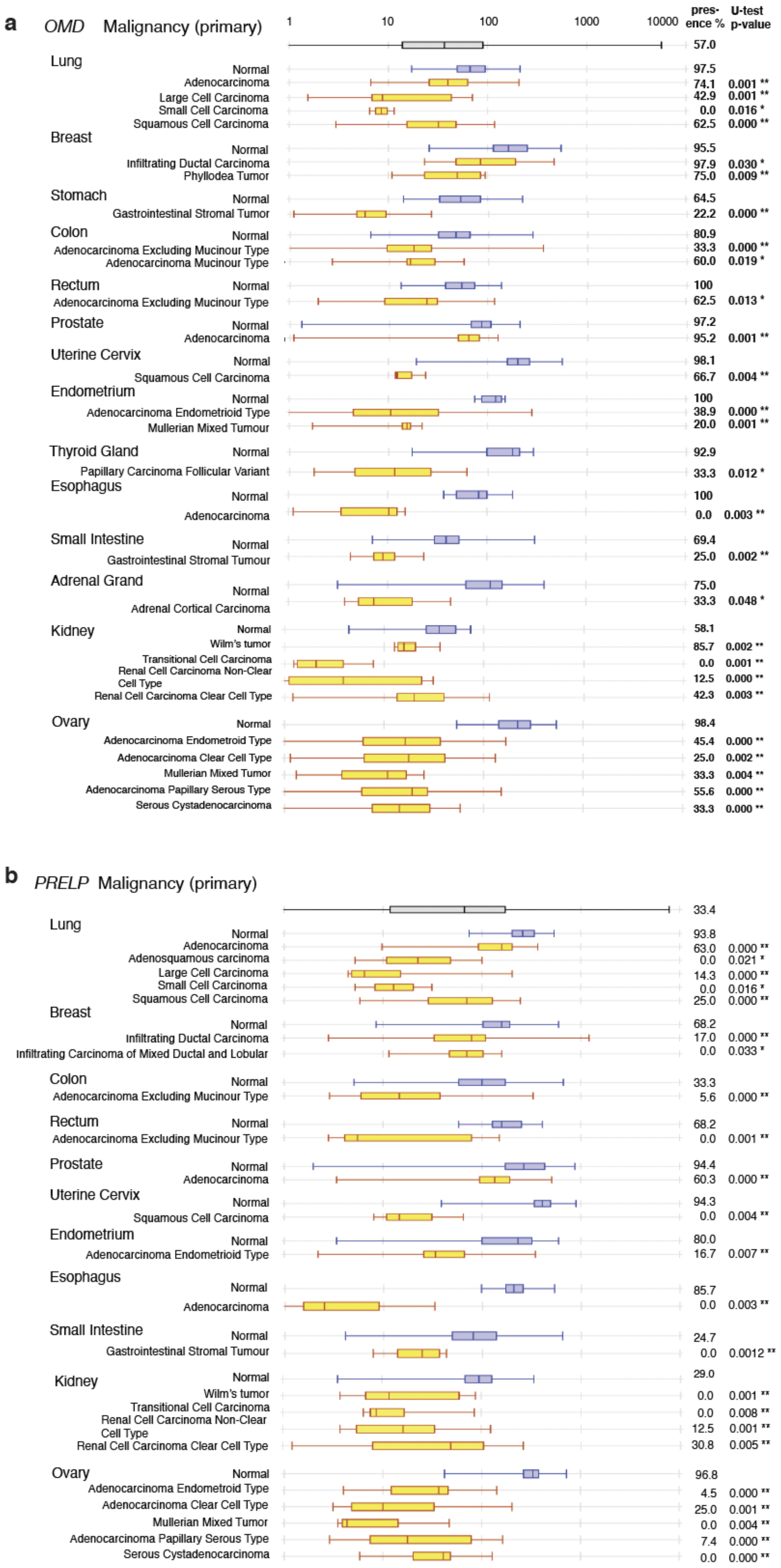

Figure S1. Microarray analysis of OMD and PRELP expression in various cancers and normal tissues. (**a**) Expression levels of OMD. (**b**) Expression levels of PRELP. Plots show mRNA concentration cancer samples and control normal tissues. Box plots show minimum, first

quartile, median, third quartile, and maximum for each dataset. *, ** indicate $p < 0.05$, $p < 0.01$, respectively.

a

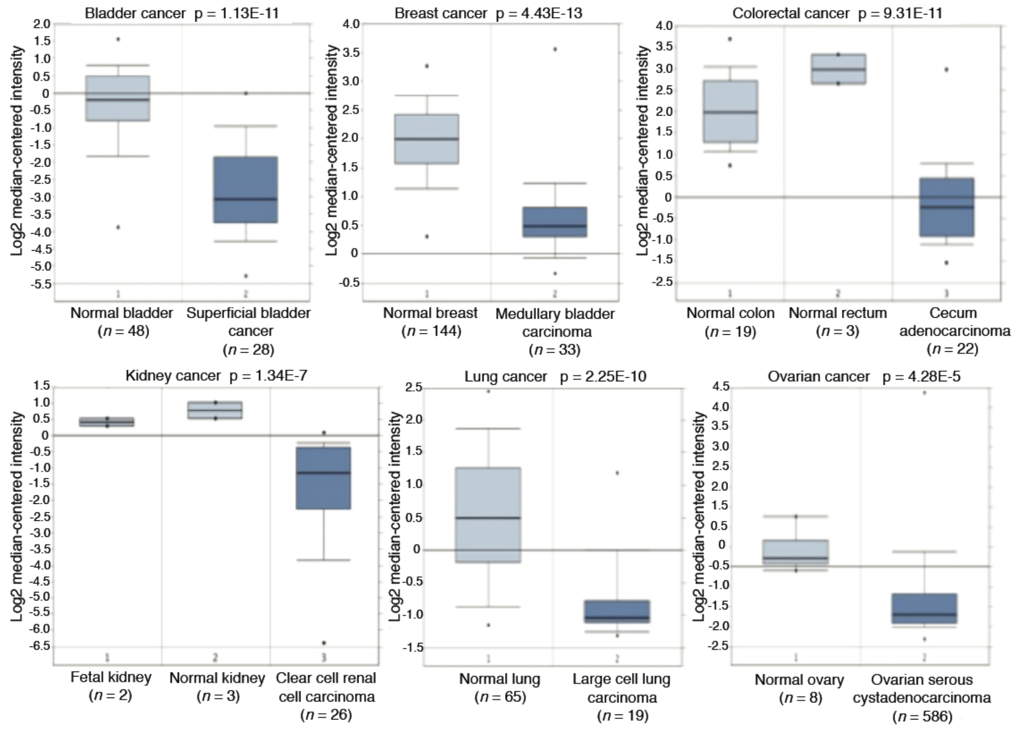

b

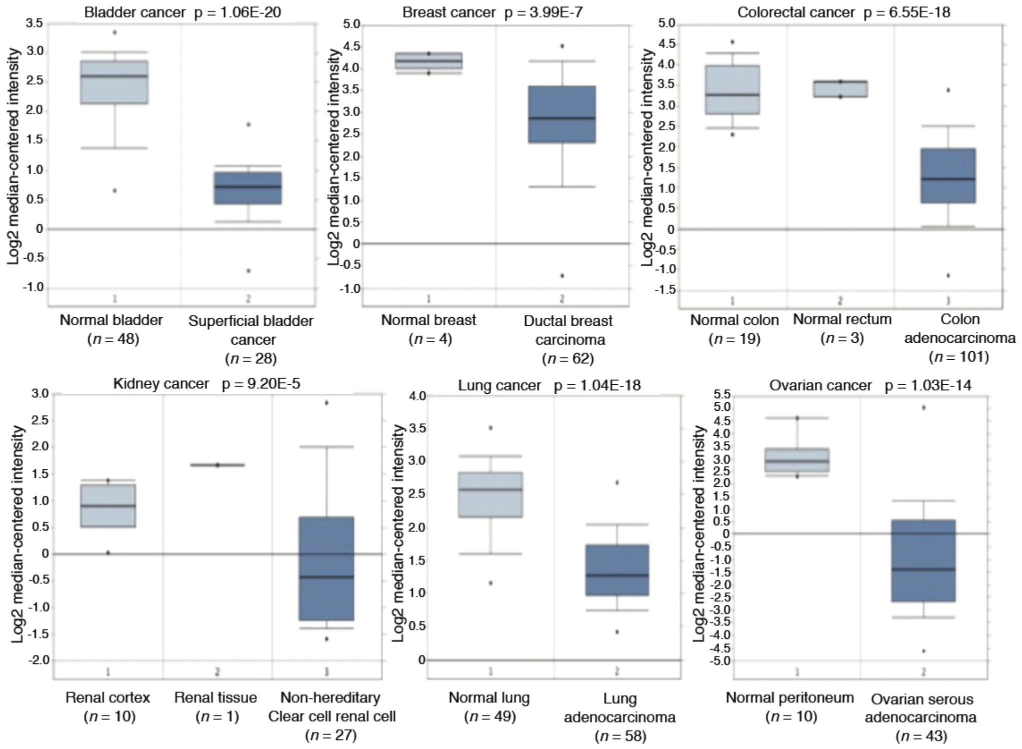

Figure S2. Gene Logic Inc data analysis. RNA was prepared and gene expression analysis was determined at Gene Logic Inc. using Affymetrix GeneChip® HG-U133Plus2 microarrays containing oligodeoxynucleotides that correspond to approximately 40,000 genes/ESTs. ((**a**) Expression levels of OMD. (**b**) Expression levels of PRELP. * indicate $p < 0.05$.

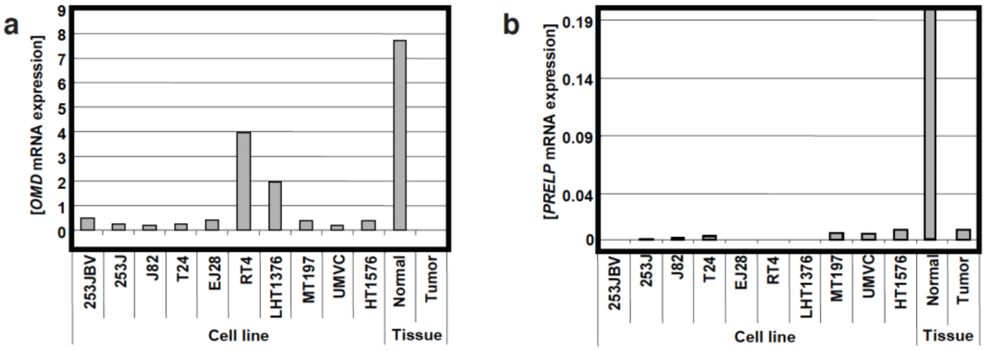

c Cancer OMD overexpression

Ingenuity Canonical Pathways	-log(p-value)
p53 Signaling	1.89
Myc Mediated Apoptosis Signaling	1.83
Molecular Mechanisms of Cancer	1.57
Renal Cell Carcinoma Signaling	1.55
HER-2 Signaling in Breast Cancer	1.30

d Cancer PRELP overexpression

Ingenuity Canonical Pathways	-log(p-value)
Molecular Mechanisms of Cancer	3.72
Hereditary Breast Cancer Signaling	2.73
Prostate Cancer Signaling	2.58
p53 Signaling	2.20
Pancreatic Adenocarcinoma Signaling	2.15
Polyamine Regulation in Colon Cancer	1.95
FAT10 Cancer Signaling Pathway	1.75

e Cancer OMD depletion

Ingenuity Canonical Pathways	-log(p-value)
Role of Tissue Factor in Cancer	5.23
Molecular Mechanisms of Cancer	4.73
Non-Small Cell Lung Cancer Signaling	4.13
Glioma Signaling	4.06
Small Cell Lung Cancer Signaling	3.01
Acute Myeloid Leukemia Signaling	2.56
Ovarian Cancer Signaling	2.43
p53 Signaling	2.31
Endometrial Cancer Signaling	2.02
Renal Cell Carcinoma Signaling	1.90

f Cancer PRELP depletion

Ingenuity Canonical Pathways	-log(p-value)
Molecular Mechanisms of Cancer	7.16
Pancreatic Adenocarcinoma Signaling	6.25
Glioma Signaling	5.41
Glioblastoma Multiforme Signaling	3.89
Prostate Cancer Signaling	3.85
Thyroid Cancer Signaling	3.53
Endometrial Cancer Signaling	3.52
Small Cell Lung Cancer Signaling	3.43
Role of Tissue Factor in Cancer	3.18
p53 Signaling	3.04

Figure S3. *OMD* and *PRELP* expression levels in various cancer cells and ontological analysis of expression profiling data. (**a**,**b**) Expression of *OMD* (**a**) or *PRELP* (**b**) in various bladder cancer cell lines. Analysis of cell properties under standard culture conditions. (**c**–**f**) Microarray-based expression profiling data analysis. Significantly affected cancer-related biological events after OMD overexpression (**c**), PRELP overexpression (**d**), OMD depletion (**e**), and PRELP depletion (**f**). Data were analyzed through the use of IPA (QIAGEN Inc.).

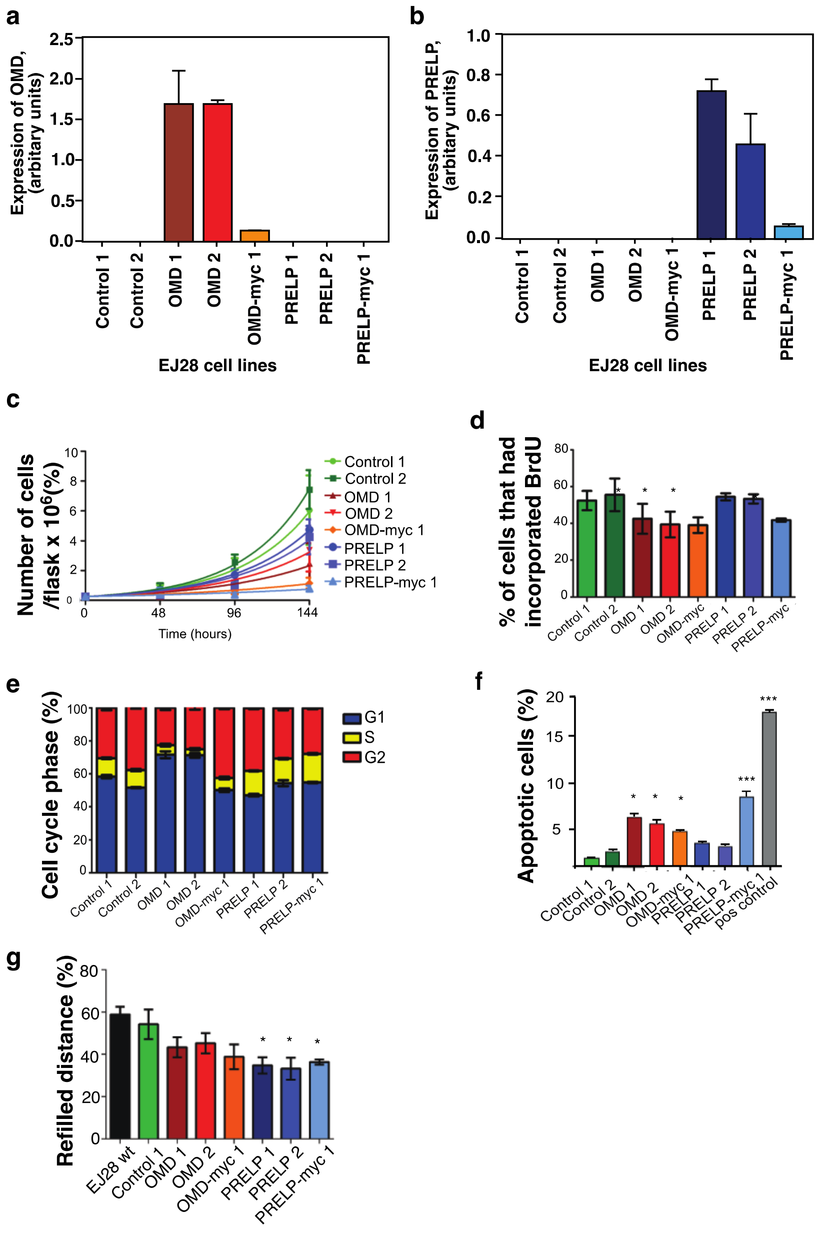

Figure S4. Effect of OMD or PRELP on cell properties under standard cell culture conditions. Expression of *OMD* and PRELP in EJ28 cells were determined by semi-quantitative RT-PCR. (**a**) Expression of *OMD*. (**b**) Expression of *PRELP*. (**c**) Cell proliferation assay by cell counting. (**d**) Proliferation assay using BrdU. (**e**) FACS analysis of cell cycle phases. (**f**) Annexin staining of apoptotic cells. (**g**) Wound-healing scratch assay for cell migration. * indicates $p < 0.05$.

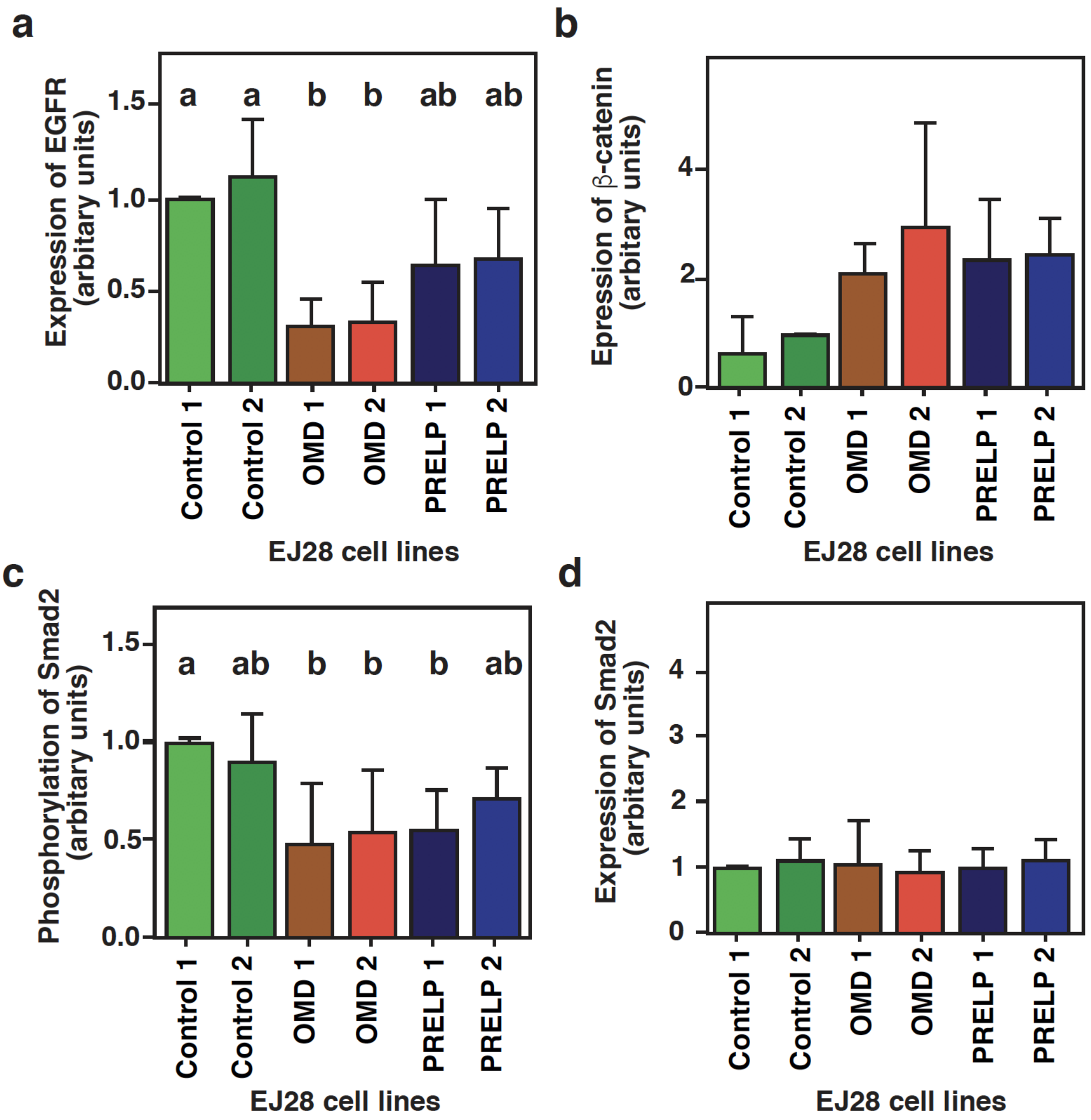

Figure S5. Quantification of OMD and PRELP effects. (**a**) Expression of EGFR was quantified after Western blotting using EGFR antibody. Statistical analysis was performed using three repeats by ANOVA ($p < 0.0001$). Error bar represents standard deviations. Newman–Keuls testing was carried out. Letter groupings, "a", "b" refer to the results of the Newman–Keuls test. Cell lines not significant different to each other are labeled with the same letter. (**b**) Expression of β-catenin was quantified. (**c**) Phosphorylated Smad2 was quantified. (**d**) Expression of Smad2 was quantified.

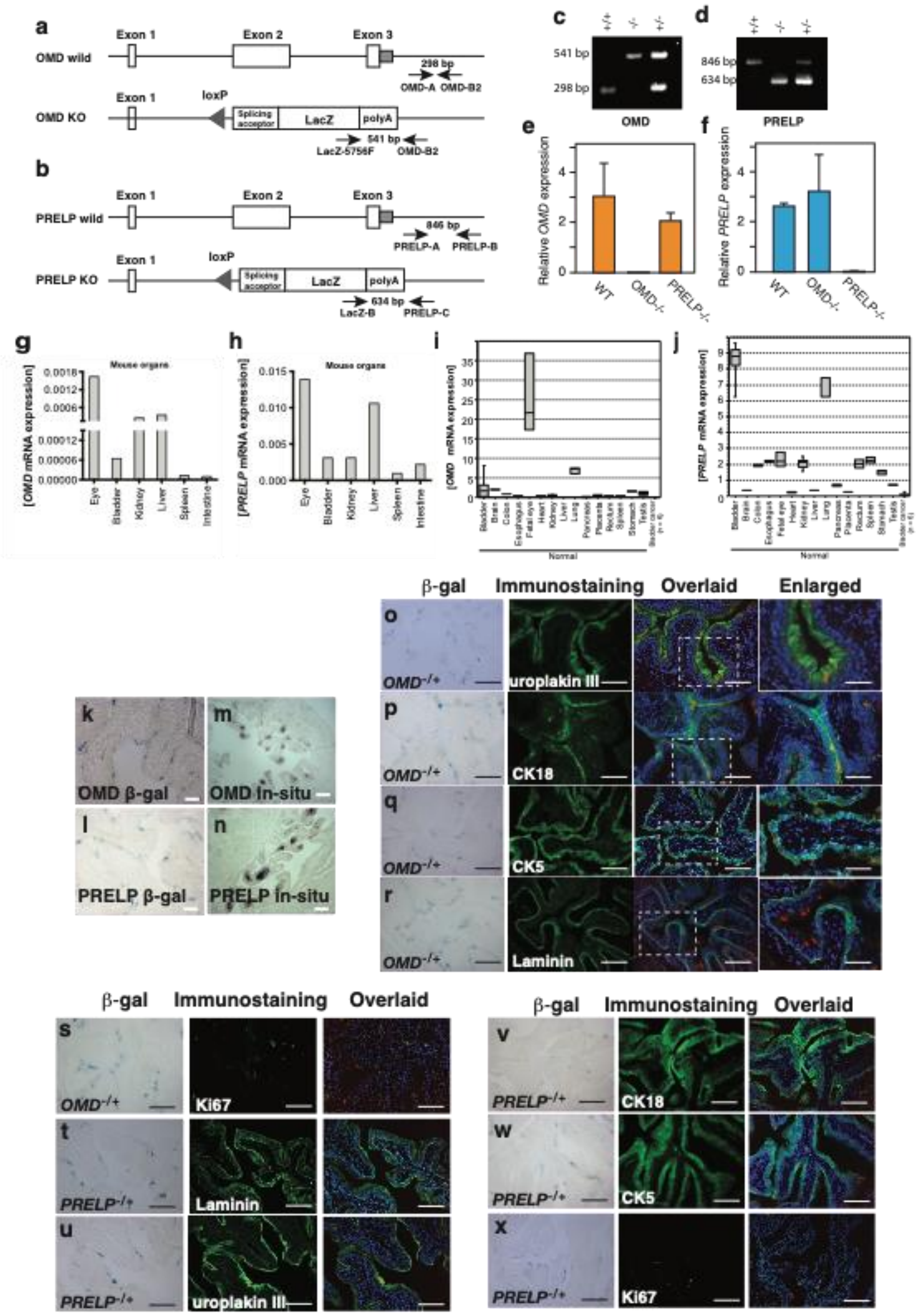

Figure S6. OMD and PRELP are expressed in subpopulation of bladder umbrella epithelial cells. (**a,b**) Construction of *OMD*$^{-/-}$ (**a**) and *PRELP*$^{-/-}$ (**b**) mice. (**c,d**) Genotyping of *OMD*$^{-/-}$ (**c**) and *PRELP*$^{-/-}$ (**d**). Primer positions are indicated in (**a**) and (**b**). (**e,f**) *OMD* and *PRELP* expression analyzed by qRT-PCR in knockout mice. (**g,h**) Expression of OMD (**g**) and PRELP (**h**) in various mouse tissues. (**i,j**) Expression of OMD (**i**) and PRELP (**j**) in various human tissues. (**k,l**) β-galactosidase staining of bladder derived from *OMD*$^{-/+}$ (**k**) and *PRELP*$^{-/+}$ (**l**). (**m,n**) In situ hybridization of *OMD* (**m**) and *PRELP* (**n**) in the *WT* bladder. (**o–r**) Double staining of *OMD*$^{-/+}$ bladder with β-galactosidase and marker antibodies. uroplakin III (**o**), CK18 (*p*), CK5

(**q**), Laminin (**r**), or Ki67 (**s**). Scale bar represents 100 μm. Each set has β-gal, antibody staining, and an overlaid image, where β-gal staining is marked by a red color, with 4′6-diamidino-2-phenylindole (DAPI), and an enlarged overlaid image in order. (**t–x**) Double staining of *PRELP*-/+ bladder with β-galactosidase and laminin (**t**), uroplakin III (**u**), CK18 (**v**), CK5 (**w**), or Ki67 (**x**) antibody. Scale bar represents 100 μm.

a In *OMD*$^{-/-}$ bladder epithelia

Ingenuity Canonical Pathways	-log(p-value)
Molecular Mechanisms of Cancer	15.2
Colorectal Cancer Metastasis Signaling	12.9
Glioblastoma Multiforme Signaling	9.63
Breast Cancer Regulation by Stathmin1	7.30
Glioma invasiveness Signaling	5.52
Endocannabinoid Cancer Inhibition Pathway	5.04
Chronic Myeloid Leukemia Signaling	5.00
Calcium-induced T Lymphocyte Apoptosis	4.83
Ovarian Cancer Signaling	4.75
FAT10 Cancer Signaling Pathway	4.66
Glioma Signaling	3.50
Thyroid Cancer Signaling	3.30
Endometrial Cancer Signaling	2.98
Prostate Cancer Signaling	2.65
Hereditary Breast Cancer Signaling	2.32
Bladder Cancer Signaling	2.17
Small Cell Lung Cancer Signaling	1.89
Cancer Drug Resistence By Drug Efflux	1.76
Renal Cell Carcinoma Signaling	1.52
Melanoma Signaling	1.51
Estrogen-Dependent Breat Cancer Signaling	1.41

b In *PRELP*$^{-/-}$ bladder epithelia

Ingenuity Canonical Pathways	-log(p-value)
Colorectal Cancer Metastasis Signaling	2.77
Endocannabinoid Cancer Inhibition Pathway	2.61
Molecular Mechanisms of Cancer	2.15
Breast Cancer Regulation by Stathmin1	1.97
Ovarian Cancer Signaling	1.94
Glioblastoma Multiforme Signaling	1.75

c

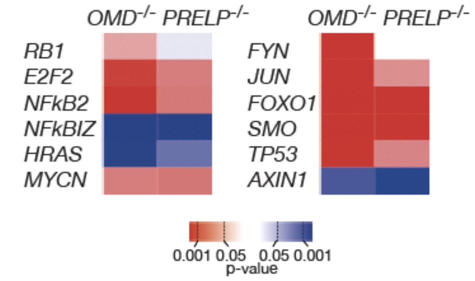

d Molecular Mechanisms of Cancer

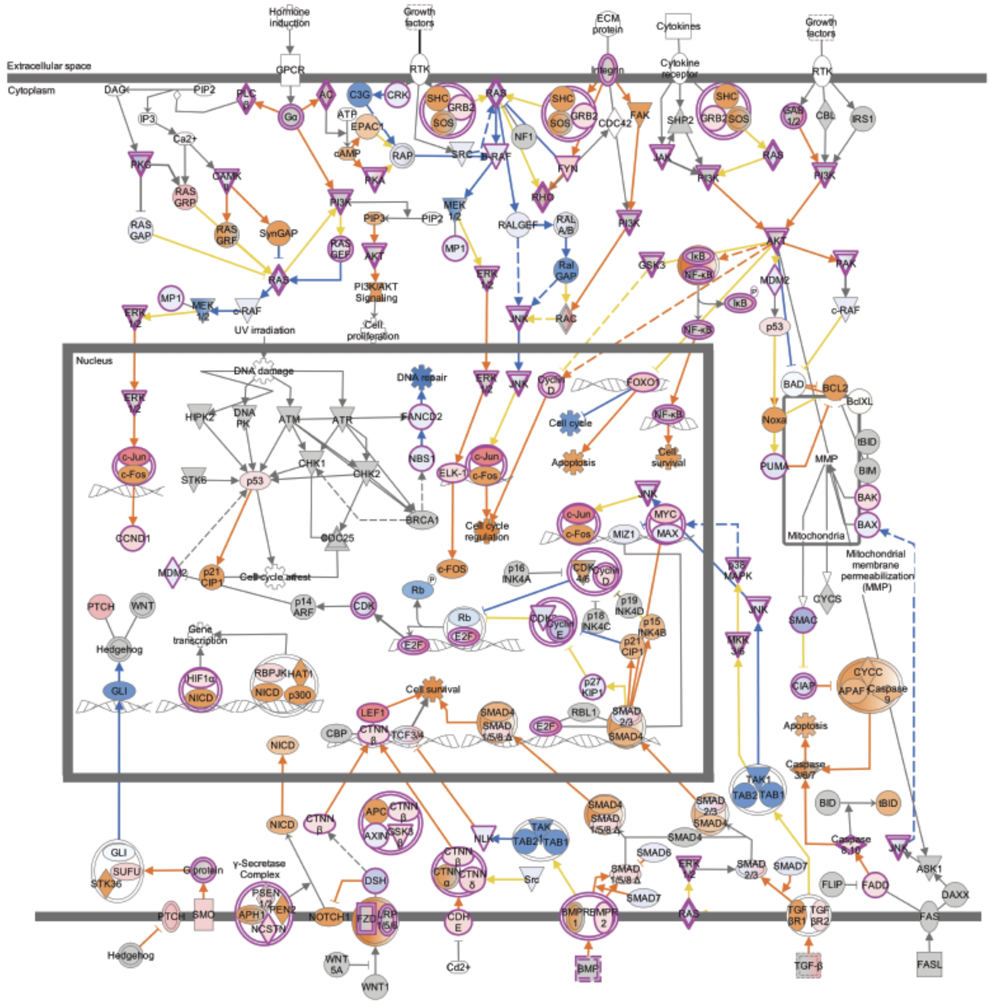

Figure S7. The ontological analysis in *OMD*−/− and *PRELP*−/− bladder epithelia. (**a,b**) Significantly affected cancer-related pathways in *OMD*−/− (**a**) and *PRELP*−/− (**b**) bladder

epithelia. (**c**) Expression of cancer-related genes. (**d**) Schematic drawing of "Molecular Mechanisms of Cancer" in *OMD*$^{-/-}$. This image was created by Ingenuity Pathway Analysis according to their rule.

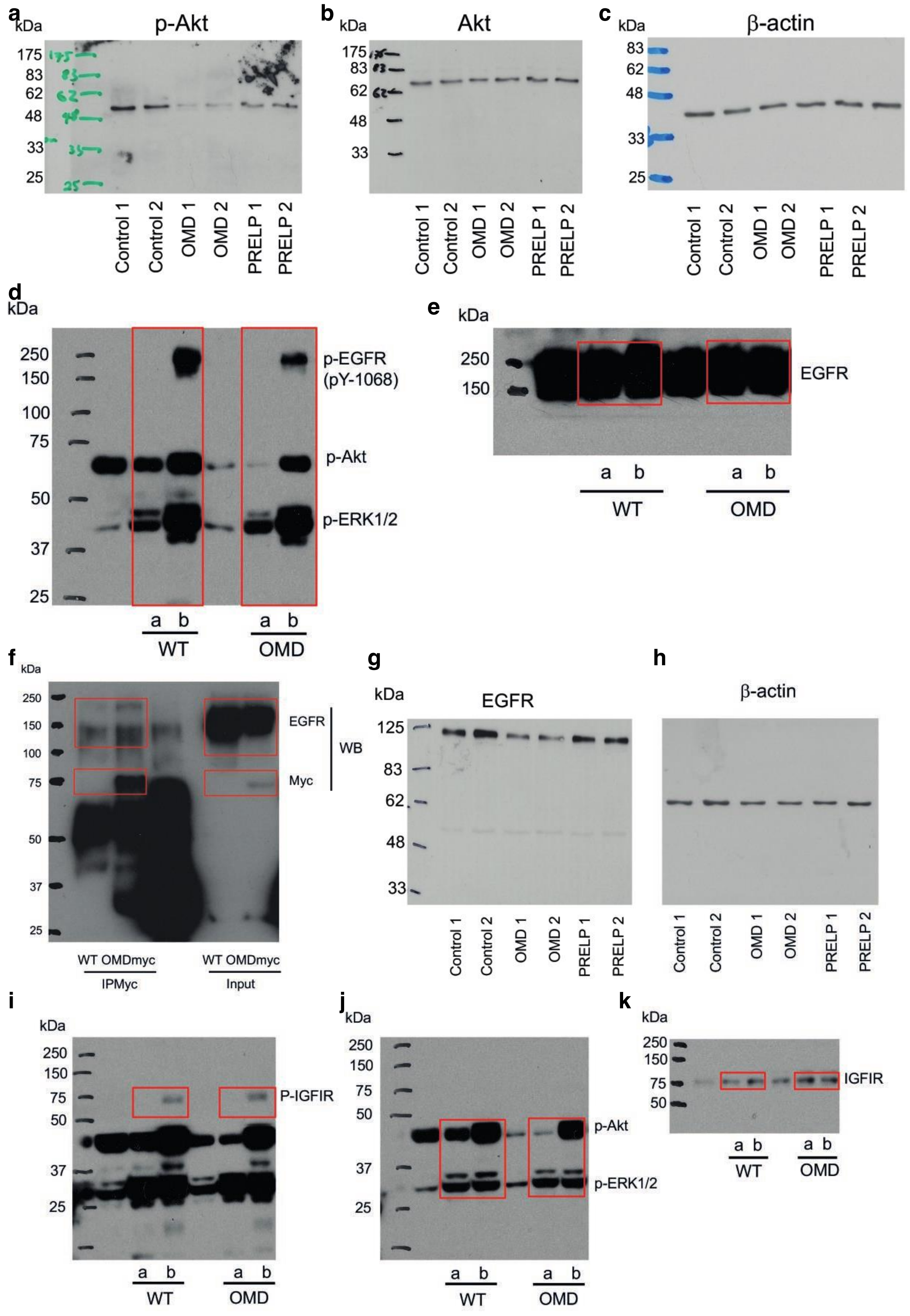

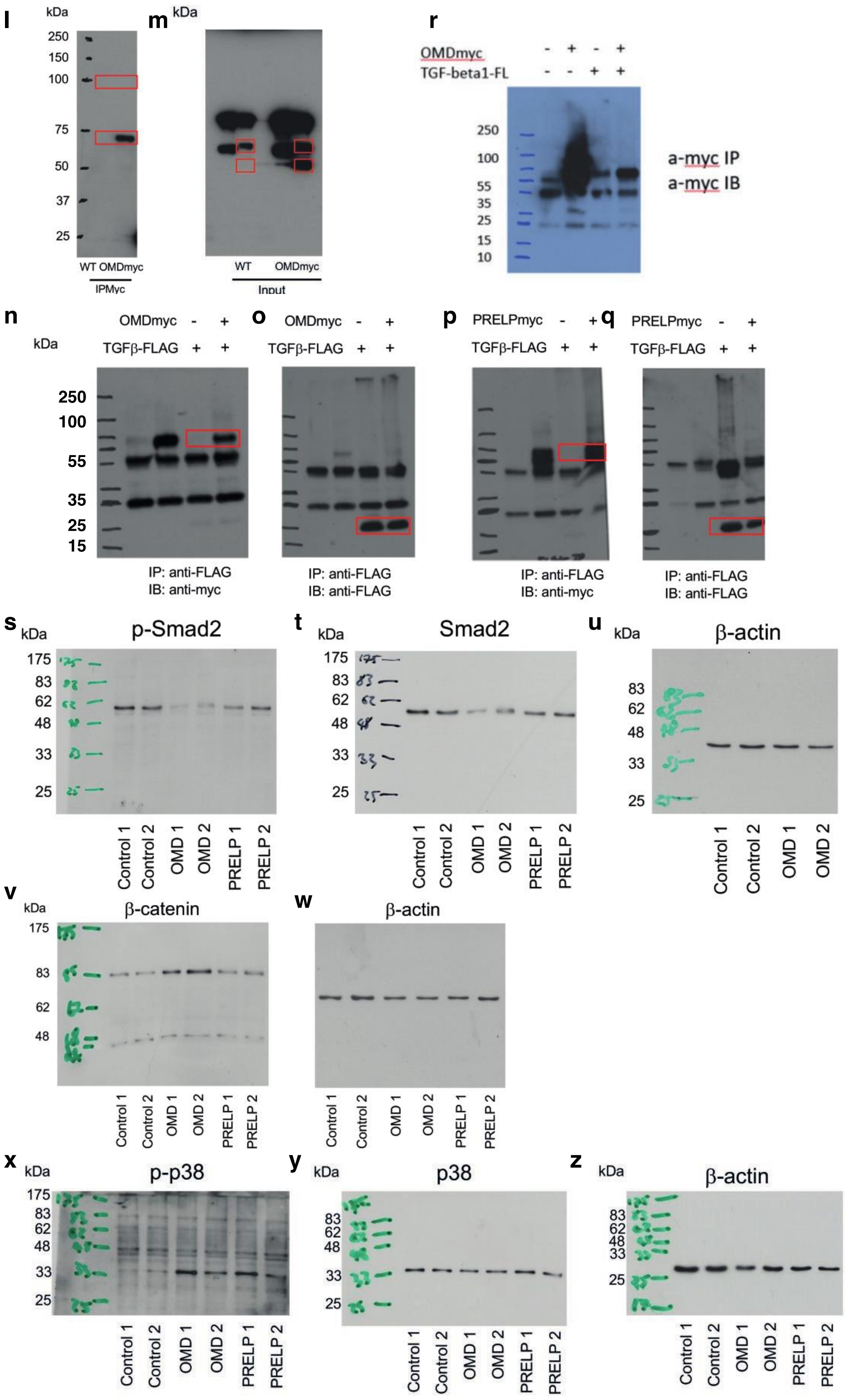

Figure S8. Original Western Blotting images used in Figure 5. (**a**) pAkt staining in Figure 5a. (**b**) Akt staining in Figure 5a. (**c**) β-actin staining in Figure 5a. (**d**) Blotted membrane was cut into three pieces based on the size. Each membrane was stained by pEGFR, pAkt, or pERK1/2 antibody and combined into one. The staining was used in Figure 5b. (**e**) EGFR staining in Figure 5b. (**f**) The membrane was cut into two. Then, each piece was staining by EGFR or myc antibody. The staining was used in Figure 5c. (**g**) EGFR staining in Figure 5d. (**h**) β-actin staining in Figure 5d. (**i**) pIGFR staining in Figure 5e. (**j**) pAkt or pERK1/2 staining in Figure 5e (**k**) IGFR staining in Figure 5e. (**l**) IGFR or myc staining after IP in Figure 5f. (**m**) IGFR or myc

staining before IP in Figure 5f. (**n**) OMD-myc. Myc staining after anti-FLAG IP in Figure 5g (**o**) OMD-myc. FLAG staining after anti-FLAG IP in Figure 5g. (**p**) PRELP-myc. Myc staining after anti-FLAG IP in Figure 5g. (**q**) PRELP-myc. FLAG staining after anti-FLAG IP in Figure 5g. (**r**) Myc staining after myc IP. (**s**) pSmad2 staining in Figure 5h. (**t**) Smad2 staining in Figure 5h. (**u**) β-actin staining in Figure 5h. (**v**) β-catenin staining in Figure 5i. (**w**) β-actin staining in Figure 5i. (**x**) pp38 staining in Figure 5j. (**y**) p38 staining in Figure 5j. (**z**) β-actin staining in Figure 5j.

© 2020 by the authors. Licensee MDPI, Basel, Switzerland. This article is an open access article distributed under the terms and conditions of the Creative Commons Attribution (CC BY) license (http://creativecommons.org/licenses/by/4.0/).
